# Developmental models of motor-evoked potential features by transcranial magnetic stimulation across age groups from childhood to adulthood

**DOI:** 10.1038/s41598-023-37775-w

**Published:** 2023-06-30

**Authors:** Dao T. A. Nguyen, Petro Julkunen, Laura Säisänen, Sara Määttä, Saara M. Rissanen, Niina Lintu, Mervi Könönen, Timo Lakka, Pasi A. Karjalainen

**Affiliations:** 1 Department of Technical Physics, University of Eastern Finland, POB 1627, 70211 Kuopio, Finland; 2 Department of Clinical Neurophysiology, Kuopio University Hospital, POB 100, 70029 KYS Kuopio, Finland; 3 Institute of Biomedicine, University of Eastern Finland, POB 162, 70211 Kuopio, Finland; 4 Department of Clinical Physiology and Nuclear Medicine, Kuopio University Hospital, POB 100, 70029 KYS Kuopio, Finland; 5 Foundation for Research in Health Exercise and Nutrition, Kuopio Research Institute of Exercise Medicine, Haapaniementie 16, 70100 Kuopio, Finland

**Keywords:** Transcranial magnetic stimulation, Electromyography - EMG, Biomedical engineering, Differentiation

## Abstract

To derive the maturation of neurophysiological processes from childhood to adulthood reflected by the change of motor-evoked potential (MEP) features. 38 participants were recruited from four groups (age mean in years [SD in months], number (males)): children (7.3 [4.2], 7(4)), preadolescents (10.3 [6.9], 10(5)), adolescents (15.3 [9.8], 11(5)), and adults (26.9 [46.2], 10(5)). The navigated transcranial magnetic stimulation was performed on both hemispheres at seven stimulation intensity (SI) levels from sub- to supra-threshold and targeted to the representative cortical area of abductor pollicis brevis muscle. MEPs were measured from three hand- and two forearm-muscles. The input–output (I/O) curves of MEP features across age groups were constructed using linear mixed-effect models. Age and SI significantly affected MEP features, whereas the stimulated side had a minor impact. MEP size and duration increased from childhood to adulthood. MEP onset- and peak-latency dropped in adolescence, particularly in hand muscles. Children had the smallest MEPs with the highest polyphasia, whereas I/O curves were similar among preadolescents, adolescents, and adults. This study illustrates some of the changing patterns of MEP features across the ages, suggesting developing patterns of neurophysiological processes activated by TMS, and to motivate studies with larger sample size.

## Introduction

Throughout developmental stages, the human neuromotor system experiences remarkable changes alongside the development of musculoskeletal structures. In a typical developmental course, children at 6 or 7-year-old can perform a full range of fundamental motor skills, such as handwriting, as their manual control and adaptation to spatial and temporal stimuli demands^1^. Then, their cognitive component of movement progressively develops alongside their rapidly growing musculoskeletal system during adolescence^1^. The motor system matures in adulthood, while it can still adapt for performance and compensate for a physical injury or the normal aging process^1^.

Underlying these changes in motor performance is the evolvement of central structures of the human motor system. The neuronal diversity, axon projection, and synapse formation are continuously reorganized to adapt to internal and external stimuli, such as hormonal, physical exercise, nutrients, and other environmental factors^1^. Developmental patterns of gray matter are regionally specific, with its thickness peaking at different timestamps in different cortical areas^2,3^. For example, the primary motor cortex (M1) is thickest around the age of nine and gradually thins out around 14^3,4^. Age-related changing patterns also include progressive myelination in both deep^5^ and superficial white matter^6,7^.

Transcranial Magnetic Stimulation (TMS) is a non-invasive brain stimulation method utilized to assess the neurophysiological functions in the corticospinal tract^8^. If the stimulus at a sufficient stimulation intensity (SI) is delivered at the representative cortical area of hand muscles, it induces a motor-evoked potential (MEP) in the contralateral hand muscles^8^. MEP amplitude increases asymptotically to SI, thus quantifying the level of corticospinal excitability^9^. The motor threshold (MT) is the threshold of inducing a motor response of a certain amplitude level with a 50% chance and is considered a measure of the network’s excitability^9^.

Other MEP features also provide the versatile characteristics of corticospinal tract function. The onset latency of MEP represents the motor conduction time for activation to travel from the stimulated area to the distal muscle, providing insightful information in the descending motor pathway^10–12^. Another essential feature of MEP is its duration, which might reflect neuronal activities in both cortical and spinal processes, including propriospinal and non-propriospinal interneurons^13,14^. Prolonged MEPs appeared, *e.g.,* in patients with multiple sclerosis, whereas shorter MEP duration has been recorded in patients with acute stroke^13^. Furthermore, polyphasia of MEPs, defined based on the number of phases and turns of the MEP waveform, is considered a potential biomarker of hyperexcitation in several movement disorders and motor neuron diseases^15,16^, and has been observed previously in children^17,18^.

TMS has been used to study the effects of aging on corticospinal excitability^17,19^ and motor pathway development^18,20–23^, with the central findings indicating that the excitability to TMS increases with aging from childhood to adulthood as the MEP amplitudes and latencies increase. This increase in excitability has been contributed to developmental stages of myelination at childhood^18,22,23^. The increase in onset latency is mainly contributed by the development in height and somewhat countered by the slower conduction velocities caused by immature myelination^22^. Previous studies have also provided some evidence of hemispheric asymmetry in corticospinal excitability in children. A clear hand preference, related to hemispheric lateralization is observed by the age of 6 years^24^, and the pattern of lateralization strengthens with increasing age^25^. The non-dominant hemisphere of healthy children has been reported to possess higher MTs than the dominant hemisphere, while the asymmetry vanishes towards adulthood^26^. This asymmetry has previously been associated with manual dexterity^21,27^ and could reflect the development of hemispheric dominance^28^.

TMS has recently been demonstrated as a successful tool in clinical assessments of younger participants, such as MT decreases with age, and lower MT is associated with better performance in manual dexterity^20,21,29–31^. Therefore, our primary aim was to explore the changes in the input–output (I/O) curve of multiple MEP features across four age groups from childhood to adulthood. For this purpose, we extracted a comprehensive set of MEP features, including its size, timing features, and morphological properties, and examined their changes as SI was adjusted. We hypothesized that these MEP features would display age and/or SI-dependent development when evaluated across the age groups providing novel insight into the maturational pattern of MEP morphology during aging from childhood to adulthood.

## Methods

### Study design

The study was designed to examine I/O curves of MEP features against SI across four age groups: children, preadolescents, adolescents, and young adults. The interested muscles were five upper extremity muscles, which all can be activated by a single stimulation performed at a representation area of abductor pollicis brevis (APB) at M1. SI was randomly chosen from sub- to supra-threshold to illustrate the I/O curve of MEP features against SI. Five morphological MEP features were extracted, then their I/O curves against SI were constructed using linear mixed models. Furthermore, the stimulation was performed on both hemispheres to investigate the effect of the stimulated side on the I/O curves.

### Participants

The present study was conducted with 38 healthy participants, categorized into four discrete age groups: children, preadolescents, adolescents, and adults (demographics in Table 1). All participants were informed about the nature of the study. After having received a detailed description of the procedure, the participants provided written informed consent to the research and to publication of the results. Consent was also provided from the legal guardian in the case of a participant being under 15 years of age. The study was approved by the Research Ethics Committee of the Hospital District of Northern Savo (48/2010), and the experiments were carried out by the latest version of the Helsinki declaration.

**Table 1:** Participant demographics including age (mean in years [SD in months]), height (mean [SD]) and resting motor threshold (rMT) as of maximum stimulator output (%) measured on the left and right hemisphere (mean [SD]).

Group	n	Sex (male)	Age (years [months])	Height (cm)	rMT (%)	
					left	right
Children	7	4	7.3 [4.2]	128.3 [2.2]	59.6 [8.0]	63.6 [5.0]
Preadolescents	10	5	10.3 [6.9]	148.1 [7.5]	49.8 [8.4]	50.5 [9.9]
Adolescents	11	5	15.3 [9.8]	169.7 [8.0]	40.5 [7.2]	38.6 [8.1]
Adults	10	5	26.9 [46.2]	170.0 [10.7]	41.6 [7.7]	40.0 [6.5]

The child and preadolescent groups were recruited from a population sample of children who participated in the Physical Activity and Nutrition in Children (PANIC) study at the Institute of Biomedicine, University of Eastern Finland^32^. Adolescents were recruited from pupils in the 8th grade from the nearest comprehensive school, and the adults were students from the University of Eastern Finland and staff of the TMS laboratory. All participants were right-handed, except for one ambidextrous but predominantly right-handed boy in the child group. The handedness was determined by the Waterloo Handedness Questionnaire (in revised and reduced form with 20 items).

### Measurements

**Figure 1:**
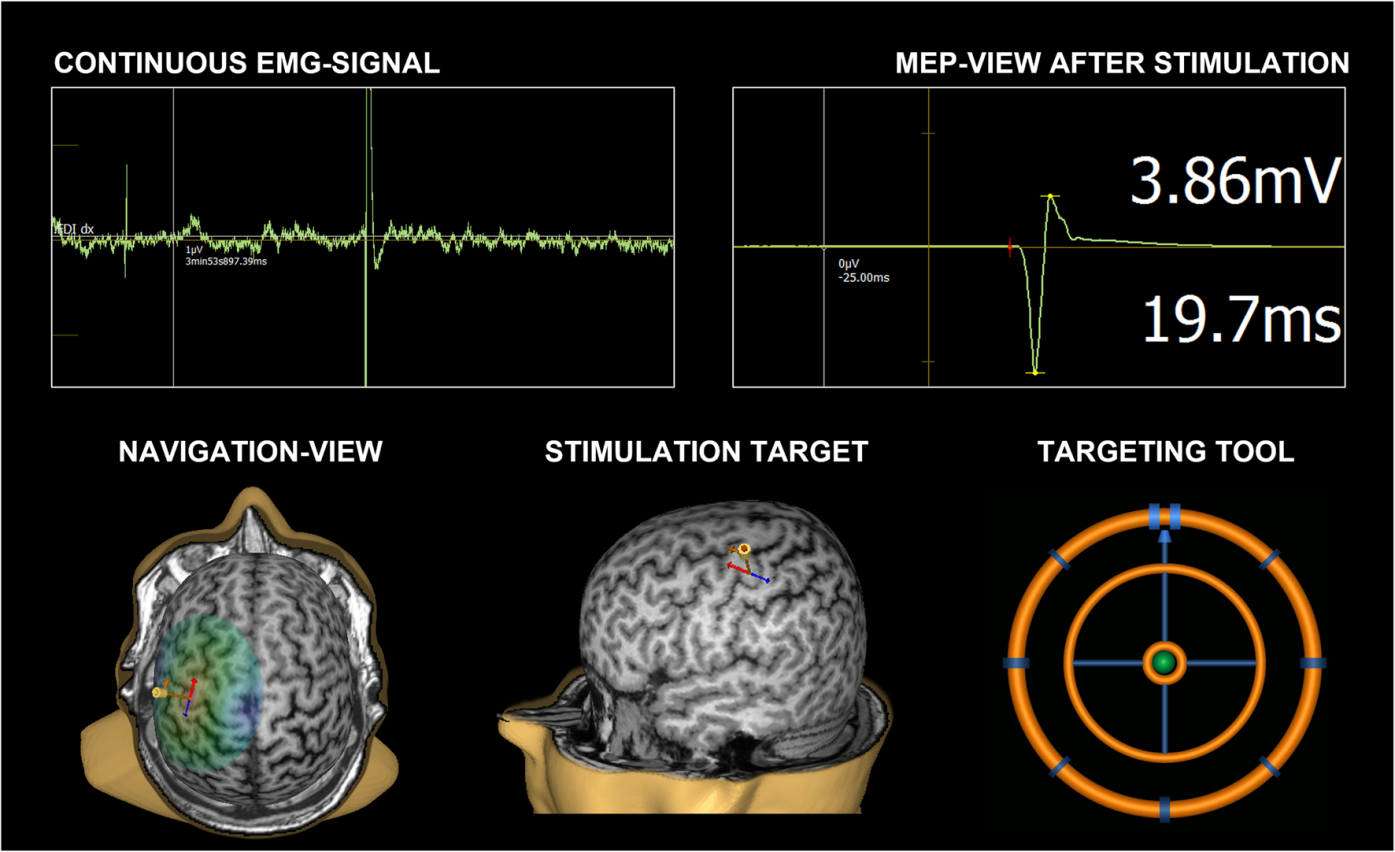
Information utilized during navigated TMS procedure. The real-time neuronavigation enables visualization and electric field estimation and maximum location display of the TMS indicating the locus of stimulation on the cortex, while also enabling synchronized continues and stimulus-locked recording of MEPs via electromyographic signal (EMG). MEP onset latency and amplitude are immediately analyzed and displayed. All stimulus locations are recorded, and any location can be repeated with the same coil configuration (all degrees-of-freedom) using the targeting tool, so that the coil is positioned identically on the scalp during repeated stimulations, e.g. when measuring resting motor threshold of I/O curve.

Structural T1-weighted image of 1 mm × 1 mm × 1 mm voxel size was obtained for each participant by a 3.0 T MRI scanner (Philips Achieva TX; Philips Healthcare, Eindhoven, The Netherlands). The experiment was performed using a TMS stimulator (eXimia version 3.2.2.; Nexstim Plc, Helsinki, Finland) with a figure-of-eight-coil and a system-integrated 6-channel electromyography (EMG) device. The navigated TMS-system included the neuronavigation software, a visual guide for the examiner to position the stimulation coil on the subject’s head in real time and to log the position and response information related to each stimulus. The software utilized a reconstructed individualized head model from the subject’s structural MRI scan, registered it to the stimulation coil’s coordination system and visualized both models in three-dimensional space (Fig. 1)^33–35^.

First, the optimal representation area, so-called the “hotspot”, of the APB muscle at M1 was located. The resting motor threshold (rMT) was determined at the hotspot using the TMS Motor Threshold Assessment Tool (MTAT version 2.0). A sequence of 70 randomized stimuli was configured in advance, in which 10 stimuli for each of seven SI levels from 90 to 150% rMT by steps of 10% rMT. The inter-trial interval was also configured to be randomly within 4–6 s^36^. The randomized sequences were generated in MATLAB (version R2019b; MathWorks Inc., Natick, Massachusetts, USA) before the sessions for all possible rMT values. The sequences of 70 stimuli associated with the rMT values of the subject were then uploaded to the stimulator for it to perform the stimulation automatically.

qIn addition to APB, the surface EMG was recorded simultaneously from the following four muscles contralateral to the stimulated hemisphere: flexor carpi radialis (FCR), extensor carpi radialis (ECR), abductor digiti minimi (ADM), and first dorsal interosseous (FDI). The stimulation was performed on both hemispheres, and EMG was recorded from contralateral muscles.

### MEP preprocessing and feature extraction

**Figure 2:**
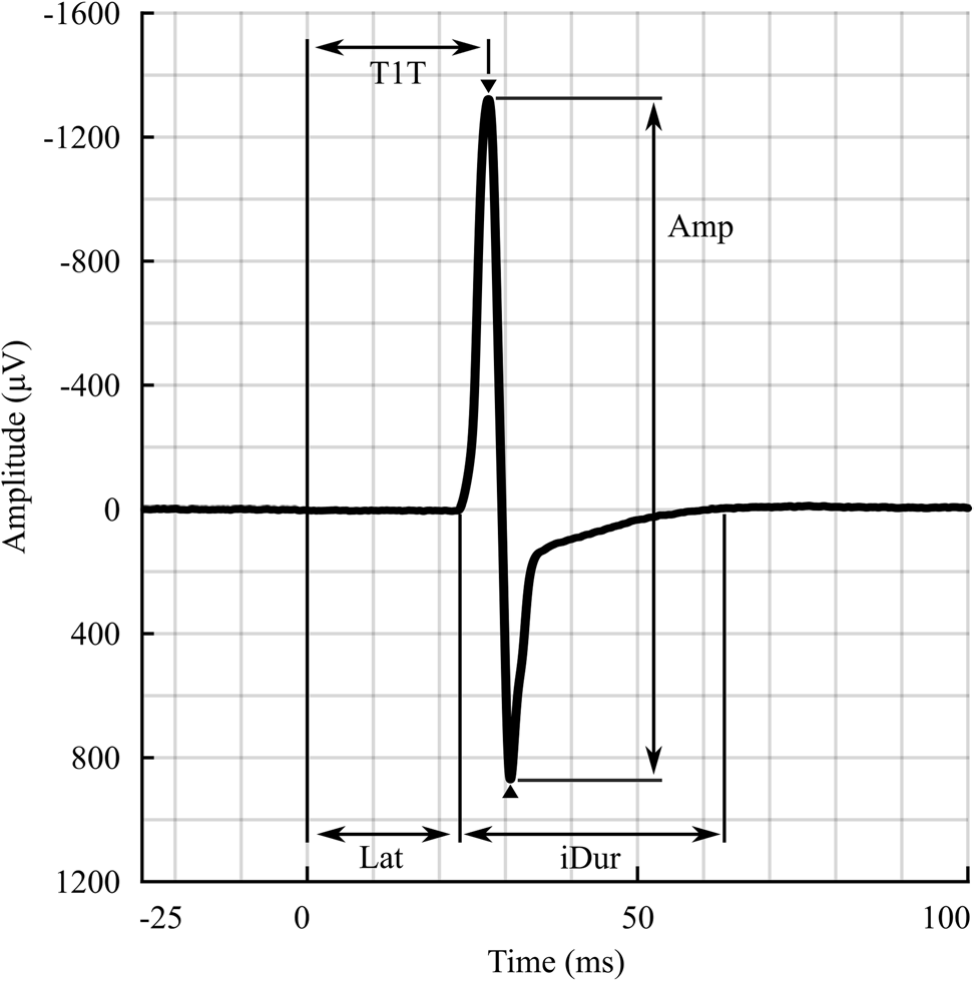
Motor-evoked potential (MEP) features. Five MEP features were included in this study: Amplitude (*Amp*), *iDur* (terminal-included duration), onset latency (*Lat*), peak latency (*T1T*) and the number of turns (*NT*) with turns indicated in the figure by (down pointed filled inverted triangle and filled triangle).

The recorded EMGs were preprocessed offline using MATLAB software, including 50 Hz noise removal and segmenting into sets of responses timing from − 50 to 150 ms regarding the stimulation time^37^. Five MEP features representing size (amplitude: *Amp*), timing point (onset latency: *Lat*, peak latency: *T1T*)), duration (terminal-included duration: *iDur*), and polyphasia (number of turns: *NT*) (Fig. 2) were studied. For more detailed descriptions, see^37^.

### Linear mixed-effect model

The linear mixed-effect (LME) models were constructed for each muscle to investigate the changing patterns of I/O curves of MEP features against SI across four age groups and the differences between two stimulated sides. Thus, this LME model contained three main factors: Age (*Age*; Child, Preadolescent, Adolescent, Adult), stimulated hemisphere (*Hemis*; Left, Right), SI (*SI*; 90, 100, 110, 120, 130, 140, 150% rMT) and their two-way interactions ($Age {\times} Hemis$, $Age\times SI$, and $Hemis\times SI$):

1 $$\begin{aligned}  x & \sim Age+SI+Hemis+\left(1|ID\right) \\ &  \quad + Age\times SI+Age\times Hemis+SI\times Hemis\end{aligned}$$

in which *x* is the MEP feature (*Amp*, *iDur*, *Lat*, *T1T*, *NT*). The term *1|ID* indicates that the subject ID was used as a random factor to determine the inter-subject variation. The three-way interaction *Age* × *SI* × *Hemis* was excluded as its effect was not statistically significant in any model.

‘lmeTest’ package (version 3.1-3) in R (version 4.0.5; R Foundation for Statistical Computing, Vienna, Austria) was used to build these LME models. The change of MEP features, as defined by the estimated marginal mean (EMM) and their 95% confidence intervals (CIs) for each factor, was obtained by ‘emmeans’ (version 1.5.5-1). The significance of each fixed effect was tested by the Type II Wald chi-square test, and reported in Cramer’s *V* for the interpretation of effect size:

2 $$\begin{array}{c}V=\sqrt{\frac{{\chi }^{2}}{n \cdot df}}\end{array}$$

in which *V* is the Cramer’s *V*, *χ*^2^ is the chi-square value, *n* is the number of samples (38), and *df* is the degree of freedom.

The random effect’s significance was tested by the maximum likelihood ratio test (LRT). The residual of each fitting was observed manually based on their histogram’s symmetry. Furthermore, the histogram, boxplot, and Spearman’s correlation coefficients of MEP features grouped by age, muscle, SI, stimulated side, and sex were depicted in Supplement Information, Figs. S1–5, respectively. The intra-subject correlation coefficients of MEP features were calculated in MATLAB.

To test whether the effect size of our analysis was sufficient, we conducted a leave-one-out cross-validation (LOOCV) test. This test excluded all MEP data from one subject at a time, and the same LME models were fitted on the remaining data. 38 iterations yielded 38 Cramér’s *V* and their corresponding *p*-values, for which we calculated 95% CIs. Then we assessed: (1) if Cramér’s *V* of the LME models performed on all samples (fullset LME) lies within 95% CIs obtained by LOOCV test, and (2) if it would not alter the effect’s significance level. If yes, the fullset analysis is not prone to the type I error.

## Results

**Figure 3:**
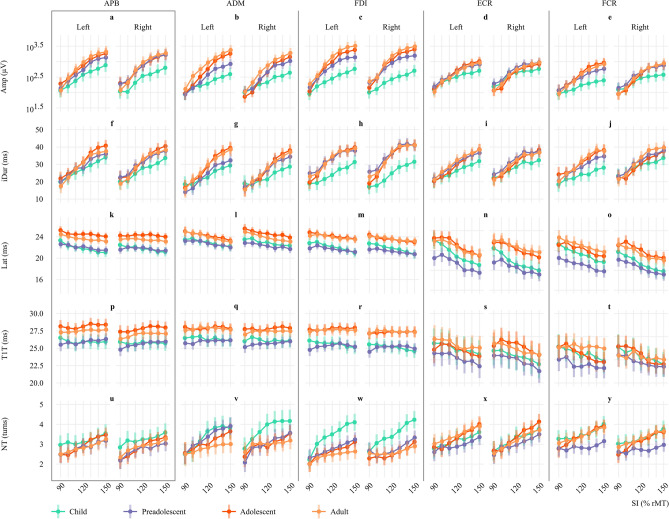
Input–output curves of MEP features against stimulation intensity (*SI*) normalized against individual resting motor threshold (rMT) in four age groups. Each subplot represents the estimated marginal mean (EMM) of MEP features and their 95% confidence interval obtained by linear mixed effect modeling. In each subplot, the EMM across four age groups was plotted separately by each muscle. The amplitude (*Amp*) is plotted in the base-10 logarithmic scale. APB, abductor pollicis brevis (the primary targeted muscle, enclosed by the grey box); ADM, abductor digiti minimi; FDI, first dorsal interosseous; ECR, extensor carpi radialis; FCR, flexor carpi radialis; *iDur*, terminal-included duration; *Lat*, onset latency; *T1T*, peak latency; *NT*, number of turns; *Hemis*, hemisphere.

**Figure 4:**
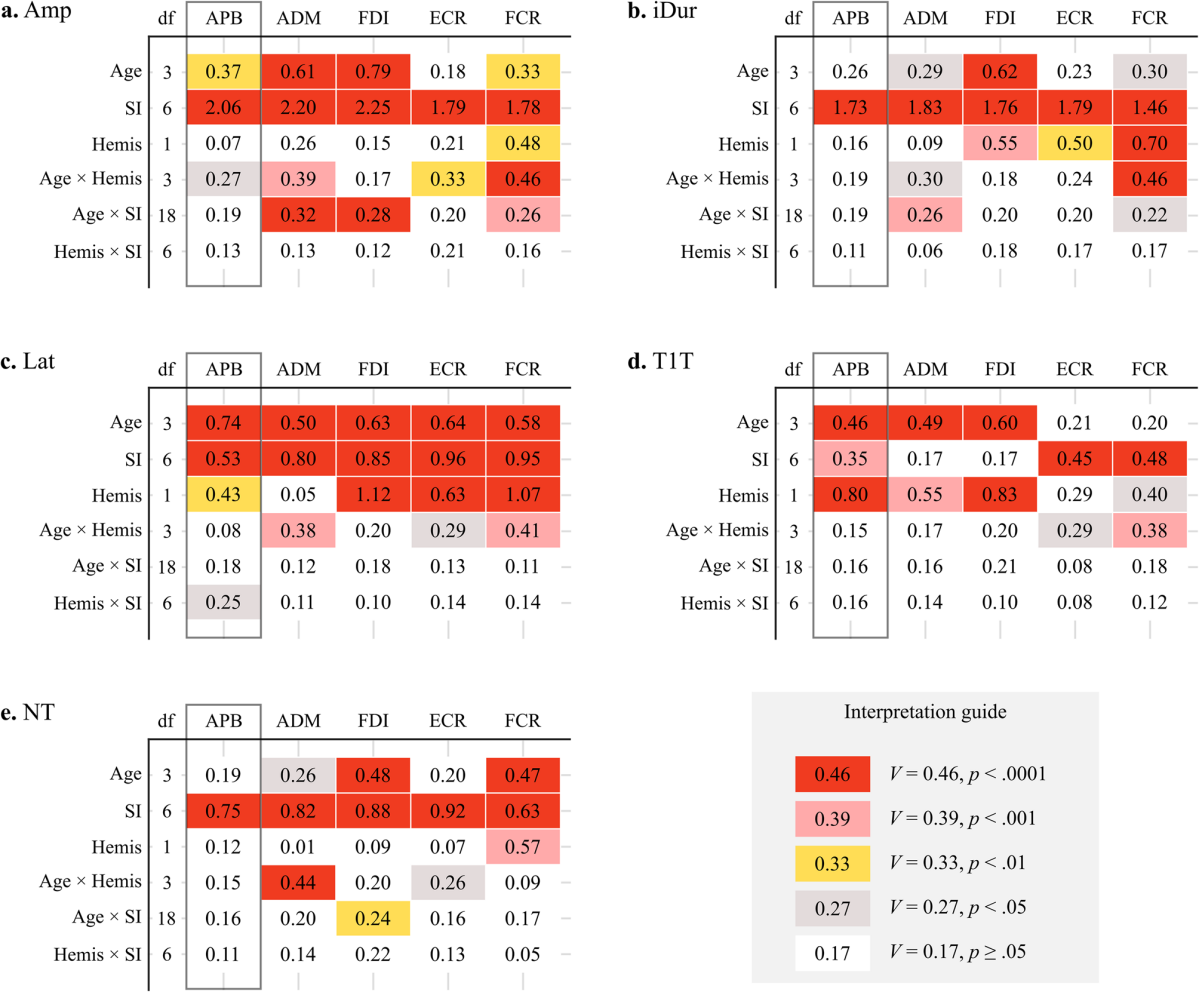
The effect of age (*Age*), stimulated hemisphere (*Hemis*), and stimulation intensity (*SI*), and their two-way interaction on MEP features. Each table represents the Cramér’s *V* value and the significance levels colored as red: *p* < 0.0001, pink: *p* < 0.001, yellow: *p* < 0.01, grey: *p* < 0.05. APB, abductor pollicis brevis (the primary targeted muscle, enclosed by the grey box); ADM, abductor digiti minimi; FDI, first dorsal interosseous; ECR, extensor carpi radialis; FCR, flexor carpi radialis; *Amp*, amplitude; *iDur*, terminal-included duration; *Lat*, onset latency; *T1T*, peak latency; NT, number of turns; *df*: degree of freedom.

The results of this study are summarized in Figs. 3 and 4. Figure 3 depicts the I/O curves of MEP features against SIs in the four age groups. Figure 4 reports the effect of each factor by the Cramér’s *V* of each effect with their significance levels colored accordingly. Detail reports on the statistical outcome from LME models are included in Supplementary Information, Table S3. Most MEP features in all muscles were significantly affected by *SI*, *χ*^2^ (6, *N* = 38) = [27.7–1151.9], *V* = [0.35–2.25]*, p* < 0.0001, except *T1T* in ADM and FDI (Fig. 4a–e). The correlations between the MEP features *Amp*, *Lat*, and *T1T* were higher as *SI* increased (Supplementary Information, Fig. S3).

### Amplitude (*Amp*) and duration (*iDur*)

In all muscles, *Amp* and *iDur* asymptotically increased with *SI* in all age groups, *χ*^2^ (6, *N* = 38) = [720.7–1151.9], *V* = [1.78–2.25], *p* < 0.0001; *χ*^2^ (6, *N* = 38) = [485.0–760.0], *V* = [1.46–1.83], *p* < 0.0001*,* respectively (Figs. 3a–j, 4a,b). *Amp* in children were the lowest compared to other groups in most muscles, except ECR (Fig. 3a–j). On the other hand, the effect of Age on *iDur* was only significant in ADM, FDI and FCR, *χ*^2^ (6, *N* = 38) = [9.5, 43.8, 10.03], *V* = [0.29, 0.62, 0.30], *p* < 0.0001.

The effect of *Age* × *SI* on *Amp* was significant in ADM, FDI and FCR, *χ*^2^ (18, *N* = 38) = [45.0–70.6], *V* = [0.26–0.32], *p* < 0.0001 (Fig. 4a)*. Hemis* and *Age* × *Hemis* displayed a significant effect on *Amp* and *iDur* of several muscles, particularly FCR’s *Amp, χ*^2^ (1, *N* = 38) = 8.9, *V* = 0.47, *p* < 0.01; χ^2^ (3, *N* = 38) = 23.7, *V* = 0.46, *p* < 0.0001*,* respectively.

### Onset latency (*Lat*) and peak latency (*T1T*)

The effect of *Age* and *SI* was significant on *Lat* of all muscles, *χ*^2^ (3, *N* = 38) = [28.1–63.0], *V* = [0.50–0.74], *p* < 0.0001; *χ*^2^ (6, *N* = 38) = [63.5–208.7], *V* = [0.53—0.96], *p* < 0.0001*,* respectively (Figs. 3k–o, 4c)*. Lat* reduced with increasing *SI,* and the decrease was less pronounced in hand muscles (Fig. 3k–m) than in forearm muscles (Fig. 3n,o). Furthermore, *Lat* in hand muscles displayed less variation than in forearm muscles (Supplementary Information, Fig. S2). *Lat* was the shortest and overlapped in children and preadolescents despite the 20 cm height difference (Fig. 3k–o). In forearm muscles, preadolescents had the shortest *Lat*. Meanwhile, *Lat* in adolescents was the longest despite being as tall as adults. The interaction *Age* × *SI* was insignificant in any muscles (Fig. 3c).

The effect of *Hemis* and *Age* × *Hemis* was statistically significant in most muscles, except ADM (Fig. 4c). However, this effect was not visually shown in Fig. 3k–o.

Like *Lat*, *T1T* of hand muscles in children and preadolescents were superimposed and shorter than adults and adolescents (Fig. 3p–r). The effect of *SI* was significant only on *T1T* of APB, *χ*^2^ (6, *N* = 38) = 27.7, *V* = 0.35, *p* < 0.001 (Figs. 3p, 4d), yet it tended to increase. The effect of *Age* on *T1T* was significant in hand muscles*, χ*^2^ (6, *N* = 38) = [24.6–40.7], *V* = [0.46–0.60], *p* < 0.0001. On the other hand, *T1T* in forearm muscles decreased with increasing *SI*, *χ*^2^ (6, *N* = 38) = [46.0, 52.5], *V* = [0.45, 0.48], *p* < 0.0001 (Figs. 3s,t, 4d). Moreover, the interaction *Age* × *SI* on *T1T* was not significant in any muscles (Fig. 4d).

### Number of turns (*NT*)

In all muscles, *NT* increased with increasing *SI* in all muscles, *χ*^2^ (6, *N* = 38) = [91.4—191.7], *V* = [0.63–0.92], *p* < 0.0001 (Fig. 4e). In hand muscles, the effect of *Age* × *SI* on *NT* was significant only in FDI, *χ*^2^ (18, *N* = 38) = 40.8, *V* = 0.24, *p* < 0.01 (Fig. 3u–w). In forearm muscles, *NT* was alike in children, adolescents, and adults, and lowest in preadolescents (Fig. 3x,y). The effect of *Age* on *NT* was only significant in FDI, and FCR, *χ*^2^ (3, *N* = 38) = [25.7, 26.6], *V* = [0.47, 0.48], *p* < 0.0001. *Age* × *Hemis* significantly affected *NT* of ADM, *χ*^2^ (3, *N* = 38) = 21.75, *V* = 0.26, *p* < 0.0001 (Fig. 4e).

### Leave-one-out cross-validation (LOOCV) test

LOOCV outputs showed that including one additional sample lead to minor change on the effect size of analyzed factors and did not lead to any change in effect’s significance level (Supplementary Information, Tables S1 and S2):

- The models using all samples only increased the effect size of two factors *Age* and *SI* slightly (by ~ 0.7% above their 95% CIs’ upper boundary) and did not affect other factors’ effect size (Supplementary Information, Table S1).
- The *p*-values remained almost unaffected (Supplementary Information, Table S2). *p*-value of the effect of *Age* on APB’s *Amp* and *iDur* became smaller, while for *Hemis* on ADM’s *NT* increased. None of these changes alters the significance level of the effect.
- One notable point is that the effect size of *Hemis* on ADM’s *NT* became smaller (*V* = 0.008, *CIs* = [0.017, 0.044]), and its *p*-value increased (*p* = 0.961, *CIs* = [0.786, 0.917]) indicating it might be affected by the small sample size. One reason is that we allowed outliers for this feature as the higher NT indicates higher complexity of MEP. The effect of Hemis in NT of this particular muscle was notable (Fig. 3v); however, it is not statistically significant with small effect size (*V* = 0.008, *p* = 0.961).

## Discussion

The overall shape of the I/O curves of MEP features was mostly similar among the four age groups. Our results confirmed the previously reported I/O curves of *Amp* and *Lat*^11^ and provided novel I/O curves for *iDur*, *T1T,* and *NT*. *Amp* of all muscles asymptotically increased with SI and saturated after the 120% rMT, consistent with the study of Devanne et al.^38^. Similar I/O curve behavior was observed in *iDur*. Instead, *Lat* decreased linearly with increasing *SI* in all four age groups. It is worth pointing out that the relation of *Amp* and *Lat* followed the sigmoid function, casting doubt on the negative correlation between these two features^11^.

Our results showed that children, the youngest investigated group, had the smallest MEPs with the shortest duration and highest polyphasia compared to other groups in all SI levels, reflecting the immaturity in their corticospinal tract. As *Amp* reflects the corticospinal excitability, this result might be due to the child–adult differences in muscle activation and muscular power, such that children are less capable of recruiting their higher-threshold, type-II motor units^39^. In addition, TMS induces activation that propagates along axons and synapses and causes secondary excitation of connected neuronal populations within local intracortical circuits and in cortico-cortical and cortico-subcortical connections, impacting neuronal activity in the targeted network (reviewed in^40^). In children, the induced electric field is wider and stronger in surrounding brain regions due to their shorter brain-scalp distance^41^ and high rMT (61.6 ± 6.7% maximum stimulator output, Table 1), which may lead to increased recruitment of early and late I-waves and consequently results in higher polyphasia. For example, if TMS pulse activates the neuronal network in ventral premotor cortex, it can induce facilitated the I2- and I3-waves through corticocortical pathway^42^. In addition, I-waves inhibition is controlled by GABAergic interneurons^43^, which is reduced in children^44^, and thus may further allow more I-waves to be recruited. On the other hand, effective connectivity of cortical motor circuits differs between children and adults, that the cortical excitability and signal spreading increases with development^29^. In a recent study, the fine motor control in under-50 g mouses is associated with a relative broadening networks of cortical motor neurons^45^, which in turns increase the propensity of inducing I-waves in the motor neuronal network. The short-range connections, which is abundant in child brain, reduces during development, while the long-range connections are strengthened^46,47^. Considering the complexity of I-waves origin, focal studies are needed to postulate the I-wave generation and modulation in children.

MEP timing features, including *Lat* and *T1T,* overlapped between children and preadolescents despite a 20 cm height difference. These features portrait the central motor conduction time, thus this finding suggests that activation potentials transmit faster in preadolescents compared to children. This result is associated with myelination of corticospinal tract during preadolescence, and aligns with the movement control advancing through early school years, at which basic motor skills are combined into more sophisticated tasks, such as handwriting and drawing^48^. Functional connectivity, such as cortico-cortical and subcortico-cortical connections, is crucial to the concurrent activity in the targeted cortical area during the stimulation. Functional connectivity shifts from short- to long-range with age in children from 6 to 10 years of age^46^. Also, ‘default’ regions, which have decreased neural activity during goal-oriented tasks, are only partially connected at early school ages, and become interconnected over development^47^.

In addition, *Lat* and *T1T* were longer in adolescents compared to those in adults despite their same height. This indicates that the motor system has not yet caught up with the dynamic hormonal changes and the quickly growing body during adolescence and becomes more efficient later in early adulthood. In addition, major cortical reorganization in their motor neuronal system, such as synaptic pruning, increasing in dendritic density and complexity, and ongoing myelination, is known to continue until early adulthood^5–9^. Neuroimaging studies demonstrated age-related increases in white matter that are thought to reflect progressive myelination^7–9^, whereas age-related decreases in grey matter are thought to reflect both synaptic pruning and myelination^4,5^. In addition, maturation of functional networks continues during adolescence. Cortico-cortical connectivity generally increases, while subcortico-cortical connections often decrease^46,47^. Axonal and transsynaptic spread of excitation of cortico-cortical and cortico-subcortical connections may also be maturation-dependent, potentially impacting neuronal activity in the targeted network and MEPs. *Lat* and *T1T* are also associated with the progression of apparent grey-matter density in the corticospinal tract through life: it increases from half of the mature size at the age of seven to the highest in adolescence and gradually decreases during adulthood^49^.

On the other hand, the models of MEP amplitude, duration, and polyphasia exhibited overlap among preadolescents, adolescents, and adults. This suggests that the developmental milestone of these features occur during childhood and become stable in preadolescents.

Unlike *Lat*, *T1T* in hand muscles of adults remained constant despite the SI variation. As *T1T* is the time point in MEP that *Amp* is the largest, its consistency against SI might refer to the precision of the motor skills in hand muscles, a crucial feature in timed motor performance^48^. This can be understood that the MEP size is proportional to SI, but the timing of an action is required to be precise.

Our results indicated only minor muscle-specific effects regarding the side of stimulation on any MEP features across the four studied groups from childhood to adulthood. All, except one ambidextrous participant, were right-handed. Hand preference already emerges over infancy, where the dominance side is shown clearly through individual–environment interactions^50^. Nonetheless, hand performance is specific for a particular task, as the motor system evolves along with the daily life activities of each individual, such as playing games, professional sports, and working^51–53^. The effect of *Hemis* and *Age* × *Hemis* was considerable in several features recorded on different muscles (Fig. 4), yet they weren’t shown clearly in the corresponding I/O curves depicted in Fig. 3. The results also suggested that this interaction could arise due to developing hemispheric dominance, albeit our sample-size and data do not enable us to comprehensively analyze this. It appears that there may also be muscle-dependent trends arising during musculoskeletal development that leads to the interhemispheric asymmetry in MEP features. As an example, strong hemispheric effect was observed in the hand muscles in case of *T1T* but not in the forearm muscles, while the effect of the interaction *Age* × *Hemis* on *T1T* was significant in forearm muscles. Indications of interhemispheric differences in corticospinal excitability and inhibition with development have been provided previously^26^. Overall, the previously reported effects of hand dominance on MEPs and rMT in the past literature have been confounding, while weak indications of hand dominance affecting the motor cortical excitability and the generated MEP have sometimes been reported^21,26,54–56^. Therefore, the side of stimulation has only minor effect on studied MEP features, with or without the interaction with other factors.

*Lat* in this study was automatically calculated^21^ and included some ripples before the MEP onset if they were significantly higher than the background activity. These ripples often occurred in preadolescents leading to a shorter *Lat* than^21^. The MEP onset in this study was marked at the baseline-crossing point by an automated algorithm. In^21^, the MEP onset was manually marked based on the visual appearance of the response at the zero-crossing point that appeared before the major peak.

### Study limitation

Even though the saturation of I/O curves of *Amp* in ECR and FCR can be achieved at SI of 120% rMT (Fig. 3), it should be noted that the stimulation was targeted to the hotspot of APB, and the representation area of ECR and FCR might be distant from this hotspot. Furthermore, gender can be a potential factor that affects MEP features, particularly during preadolescence and adolescence, when the motor system undergoes remarkable changes. However, the gender factor is not included in this study due to the insufficient sample size.

As this study is the first to investigate a comprehensive set of MEP features in a small study population, we acknowledged that the number of participants within each age-group might not cover the characteristics for the general population. Therefore, we performed LOOCV test to verify that the findings are not significantly affected by any individual’s data. LOOCV outputs indicated that the conclusions based on the mixed model analysis remained unaffected even with the smaller population. To account for potentially underpowered statistics, larger sample-size analyses are warranted to enable more detailed investigation, e.g., on the development of hand dominance through MEP features across multiple muscles.

In addition, further studies targeting a particular age group via additional stimulation paradigms are higher recommended, especially from childhood to adolescence, when the cortical reorganization undergoes dynamics changes, such as synaptic pruning, dendritic density and complexity, progressive myelination, along with the quickly growing body and hormonal rush during puberty.

## Conclusion

A thorough understanding of how the human motor system changes from the neurophysiological aspect is crucial for interpreting motor development from childhood to adulthood. Our study is the first to construct the developmental motor recruitment models of five essential MEP features from childhood to adulthood in several upper extremity muscles. Thus, the described MEP features provide essential resources in deriving the mechanism of neurophysiological circuits activated by TMS and contributes to the designing of experimental TMS protocols in different age groups, also adding to the current knowledge of motor development from childhood to adulthood.

## Supplementary Information

Supplementary Information.

## Data Availability

The datasets generated during and/or analyzed during the current study are available from the corresponding author on reasonable request.

## Abbreviations

ADM: Abductor digiti minimi
Amp: Amplitude
APB: Abductor pollicis brevis
CI: Confidence interval
ECR: Extensor carpi radialis
EMG: Electromyography
EMM: Estimated marginal mean
FCR: Flexor carpi radialis
FDI: First dorsal interosseous
I/O curve: Input–output curve
iDur: Terminal-included duration
Lat: Onset latency
LME: Linear mixed-effect model
LRT: Likelihood ratio test
M1: Primary motor cortex
MEP: Motor-evoked potential
MT: Motor threshold
NT: Number of turns
rMT: Resting motor threshold
SI: Stimulation intensity
T1T: The timing of the first turn
TMS: Transcranial magnetic stimulation

## References

[1] Payne, V. G., Block, M. E. & Yan, J. H. Understanding human motor development: The mountain metaphor. In *Human Motor Development in Individuals with and Without Disabilities* 3–16 (Nova Science Publishers, Inc, 2010).

[2] Lenroot RK, Giedd JN (2006). Brain development in children and adolescents: Insights from anatomical magnetic resonance imaging. Neurosci. Biobehav. Rev..

[3] Shaw P (2008). Neurodevelopmental trajectories of the human cerebral cortex. J. Neurosci..

[4] Vandekar SN (2015). Topologically dissociable patterns of development of the human cerebral cortex. J. Neurosci..

[5] Mabbott DJ, Noseworthy M, Bouffet E, Laughlin S, Rockel C (2006). White matter growth as a mechanism of cognitive development in children. Neuroimage.

[6] Williamson JM, Lyons DA (2018). Myelin dynamics throughout life: An ever-changing landscape?. Front. Cell. Neurosci..

[7] Norbom LB (2019). Probing brain developmental patterns of myelination and associations with psychopathology in youths using gray/white matter contrast. Biol. Psychiatry.

[8] Valero-Cabré A, Amengual JL, Stengel C, Pascual-Leone A, Coubard OA (2017). Transcranial magnetic stimulation in basic and clinical neuroscience: A comprehensive review of fundamental principles and novel insights. Neurosci. Biobehav. Rev..

[9] Rossini PM (2015). Non-invasive electrical and magnetic stimulation of the brain, spinal cord, roots and peripheral nerves: Basic principles and procedures for routine clinical and research application. An updated report from an I.F.C.N. Committee. Clin. Neurophysiol..

[10] Picht T (2012). Assessing the functional status of the motor system in brain tumor patients using transcranial magnetic stimulation. Acta Neurochir..

[11] Kallioniemi E, Pitkänen M, Säisänen L, Julkunen P (2015). Onset latency of motor evoked potentials in motor cortical mapping with neuronavigated transcranial magnetic stimulation. Open Neurol. J..

[12] Rothwell JC (1997). Techniques and mechanisms of action of transcranial stimulation of the human motor cortex. J. Neurosci. Methods.

[13] Brum M, Cabib C, Valls-Solé J (2016). Clinical value of the assessment of changes in MEP duration with voluntary contraction. Front. Neurosci..

[14] Mehdi A J van den B (2017). Physiological processes influencing motor-evoked potential duration with voluntary contraction. J. Neurophysiol..

[15] Chowdhury FA (2015). Motor evoked potential polyphasia: A novel endophenotype of idiopathic generalized epilepsy. Neurology.

[16] Snow NJ, Wadden KP, Chaves AR, Ploughman M (2019). Transcranial magnetic stimulation as a potential biomarker in multiple sclerosis: A systematic review with recommendations for future research. Neural Plast..

[17] Müller K, Hömberg V, Aulich A, Lenard HG (1992). Magnetoelectrical stimulation of motor cortex in children with motor disturbances. Electroencephalogr. Clin. Neurophysiol. Potentials Sect..

[18] Nezu A (1997). Magnetic stimulation of motor cortex in children: Maturity of corticospinal pathway and problem of clinical application. Brain Dev..

[19] Schmidt S (2015). Nonphysiological factors in navigated TMS studies; Confounding covariates and valid intracortical estimates. Hum. Brain Map..

[20] Säisänen L (2021). Primary hand motor representation areas in healthy children, preadolescents, adolescents, and adults. Neuroimage.

[21] Säisänen L (2018). Development of corticospinal motor excitability and cortical silent period from mid-childhood to adulthood—A navigated TMS study. Neurophysiol. Clin..

[22] Eyre JA, Miller S, Ramesh V (1991). Constancy of central conduction delays during development in man: Investigation of motor and somatosensory pathways. J. Physiol..

[23] Fietzek UM (2000). Development of the corticospinal system and hand motor function: Central conduction times and motor performance tests. Dev. Med. Child Neurol..

[24] Scharoun SM, Bryden PJ (2014). Hand preference, performance abilities, and hand selection in children. Front. Psychol..

[25] Marcori AJ, Monteiro PHM, Brussolo AD, Okazaki VHA (2023). The development of hand, foot, trunk, hearing, and visual lateral preference throughout the lifespan. Neuropsychologia.

[26] Garvey MA (2003). Cortical correlates of neuromotor development in healthy children. Clin. Neurophysiol..

[27] Vallence AM, Smalley E, Drummond PD, Hammond GR (2017). Long-interval intracortical inhibition is asymmetric in young but not older adults. J. Neurophysiol..

[28] Tzourio-Mazoyer N (2016). Intra- and inter-hemispheric connectivity supporting hemispheric specialization. Res. Perspect. Neurosci..

[29] Määttä S (2017). Development of cortical motor circuits between childhood and adulthood: A navigated TMS-HdEEG study: Development of Cortical Motor Circuits. Hum. Brain Mapp..

[30] Narayana S, Papanicolaou AC, McGregor A, Boop FA, Wheless JW (2015). Clinical applications of transcranial magnetic stimulation in pediatric neurology. J. Child Neurol..

[31] Schramm S, Mehta A, Auguste KI, Tarapore PE (2021). Navigated transcranial magnetic stimulation mapping of the motor cortex for preoperative diagnostics in pediatric epilepsy. J. Neurosurg. Pediatr..

[32] Eloranta A-M (2012). Dietary factors associated with overweight and body adiposity in Finnish children aged 6–8 years: The PANIC Study. Int. J. Obes..

[33] Ruohonen J, Karhu J (2010). Navigated transcranial magnetic stimulation. Clin. Neurophysiol..

[34] Hannula H, Ilmoniemi RJ (2017). Basic principles of navigated TMS. Navig. Transcranial Magn. Stimul. Neurosurg..

[35] Julkunen P (2009). Comparison of navigated and non-navigated transcranial magnetic stimulation for motor cortex mapping, motor threshold and motor evoked potentials. Neuroimage.

[36] Julkunen P, Säisänen L, Hukkanen T, Danner N, Könönen M (2012). Does second-scale intertrial interval affect motor evoked potentials induced by single-pulse transcranial magnetic stimulation?. Brain Stimul..

[37] Nguyen DTA, Rissanen SM, Julkunen P, Kallioniemi E, Karjalainen PA (2019). Principal component regression on motor evoked potential in single-pulse transcranial magnetic stimulation. IEEE Trans. Neural Syst. Rehabil. Eng..

[38] Devanne H, Lavoie BA, Capaday C (1997). Input-output properties and gain changes in the human corticospinal pathway. Exp. Brain Res..

[39] Dotan R (2012). Child-adult differences in muscle activation—A review. Pediatr. Exerc. Sci..

[40] Siebner HR (2022). Transcranial magnetic stimulation of the brain: What is stimulated?—A consensus and critical position paper. Clin. Neurophysiol..

[41] Beauchamp MS (2011). The developmental trajectory of brain-scalp distance from birth through childhood: Implications for functional neuroimaging. PLoS ONE.

[42] Shimazu H, Maier MA, Cerri G, Kirkwood PA, Lemon RN (2004). Macaque ventral premotor cortex exerts powerful facilitation of motor cortex outputs to upper limb motoneurons. J. Neurosci..

[43] Ziemann U (2020). I-waves in motor cortex revisited. Exp. Brain Res..

[44] Walther M (2009). Maturation of inhibitory and excitatory motor cortex pathways in children. Brain Dev..

[45] Biane JS, Scanziani M, Tuszynski MH, Conner JM (2015). Motor cortex maturation is associated with reductions in recurrent connectivity among functional subpopulations and increases in intrinsic excitability. J. Neurosci..

[46] Langen CD (2018). Differential patterns of age-related cortical and subcortical functional connectivity in 6-to-10 year old children: A connectome-wide association study. Brain Behav..

[47] Fair DA (2008). The maturing architecture of the brain’s default network. Proc. Natl. Acad. Sci..

[48] Largo RH (2001). Neuromotor development from 5 to 18 years. Part 2: Associated movements. Dev. Med. Child Neurol..

[49] Hervé P-Y (2009). Handedness, motor skills and maturation of the corticospinal tract in the adolescent brain. Hum. Brain Mapp..

[50] Nelson EL (2017). Toddler hand preference trajectories predict 3-year language outcome. Dev. Psychobiol..

[51] Steenhuis RE (1999). The Relation between hand preference and hand performance: What you get depends on what you measure. Laterality.

[52] Brouwer B, Sale MV, Nordstrom MA (2001). Asymmetry of motor cortex excitability during a simple motor task: Relationships with handedness and manual performance. Exp. Brain Res..

[53] Christiansen L, Larsen MN, Grey MJ, Nielsen JB, Lundbye-Jensen J (2017). Long-term progressive motor skill training enhances corticospinal excitability for the ipsilateral hemisphere and motor performance of the untrained hand. Eur. J. Neurosci..

[54] Säisänen L (2008). Motor potentials evoked by navigated transcranial magnetic stimulation in healthy subjects. J. Clin. Neurophysiol..

[55] Cueva AS (2016). Normative data of cortical excitability measurements obtained by transcranial magnetic stimulation in healthy subjects. Neurophysiol. Clin..

[56] De Gennaro L (2004). Handedness is mainly associated with an asymmetry of corticospinal excitability and not of transcallosal inhibition. Clin. Neurophysiol..

