# Supplementary material for: Developmental models of motor-evoked potential features by transcranial magnetic stimulation across age groups from childhood to adulthood
Source: Sci Rep. 2023 Jun 30;13:10604. doi: 10.1038/s41598-023-37775-w (PMC10313665; doi:10.1038/s41598-023-37775-w)
Supplement: Supplementary file 1 — Supplementary Information. [file 41598_2023_37775_MOESM1_ESM.docx]

**Title Page**

Developmental Models of Motor-evoked Potential Features by Transcranial Magnetic Stimulation across Age Groups from Childhood to Adulthood

Dao T. A. Nguyen^a, *^, Petro Julkunen^a, b^, Laura Säisänen^a,b^, Sara Määttä^b^, Saara M. Rissanen^a^, Niina Lintu^c^, Mervi Könönen^a^, Timo Lakka^c,d,e^, and Pasi A. Karjalainen^a^

^a^ Department of Technical Physics, University of Eastern Finland, POB 1627, FI-70211, Kuopio, Finland

^b^ Department of Clinical Neurophysiology, Kuopio University Hospital, POB 100, FI-70029 KYS, Kuopio, Finland

^c^ Institute of Biomedicine, University of Eastern Finland, POB162, FI-70211, Kuopio, Finland

^d^ Department of Clinical Physiology and Nuclear Medicine, Kuopio University Hospital, POB 100, FI-70029 KYS, Kuopio, Finland

^e^ Foundation for Research in Health Exercise and Nutrition, Kuopio Research Institute of Exercise Medicine, Haapaniementie 16, FI-70100, Kuopio, Finland

Corresponding author *: Dao T. A. Nguyen, Department of Technical Physics, University of Eastern Finland, POB 1627, FI-70211, Kuopio, Finland. E‑mail address: thi.dao.nguyen@uef.fi, or nguyen.dao.bk@gmail.com.

E-mail of contributing authors: Petro Julkunen (petro.julkunen@uef.fi), Laura Säisänen (laura.saisanen@pshyvinvointialue.fi), Sara Määttä (sara.maatta@pshyvinvointialue.fi), Saara M. Rissanen (saara.rissanen@uef.fi), Niina Lintu (niina.lintu@uef.fi), Mervi Könönen (mervi.kononen@pshyvinvointialue.fi), Timo Lakka (timo.lakka@uef.fi), and Pasi A. Karjalainen (pasi.karjalainen@uef.fi).


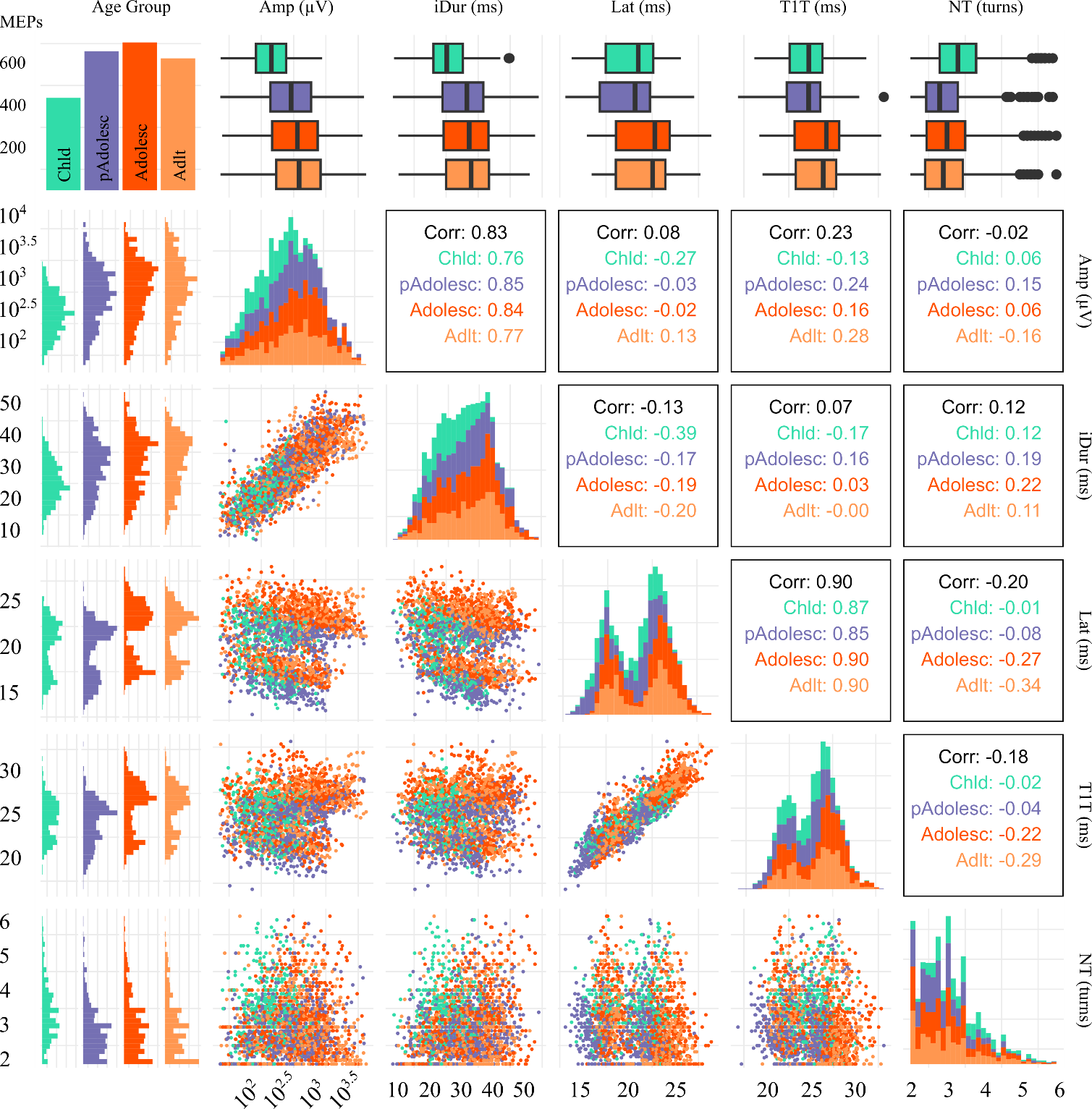


Figure S1: Feature histogram and inter-feature relationships grouped by age groups (Child: green, Preadolescent: purple, Adolescent: red, Adults: orange). The box at the top-left corner depicts the sample size at each age group, the top-most row contains boxplots of each feature, and the left-most column contains feature histograms; all are colored according to the corresponding age group. Boxes at the diagonal show feature histograms plotted against each group. Boxes above the diagonal to the right contain the correlation coefficients among features. Boxes below the diagonal to the left show scatter plots of each pair of features. Amp, amplitude; iDur, terminal-included duration; Lat, latency; T1T, timing of the first significant turn; NT, number of turns; Chld, child; pAdolesc, preadolescent; Adolesc, adolescent; Adlt, adult; Cor, Correlation coefficient; MEP, motor-evoked potential.


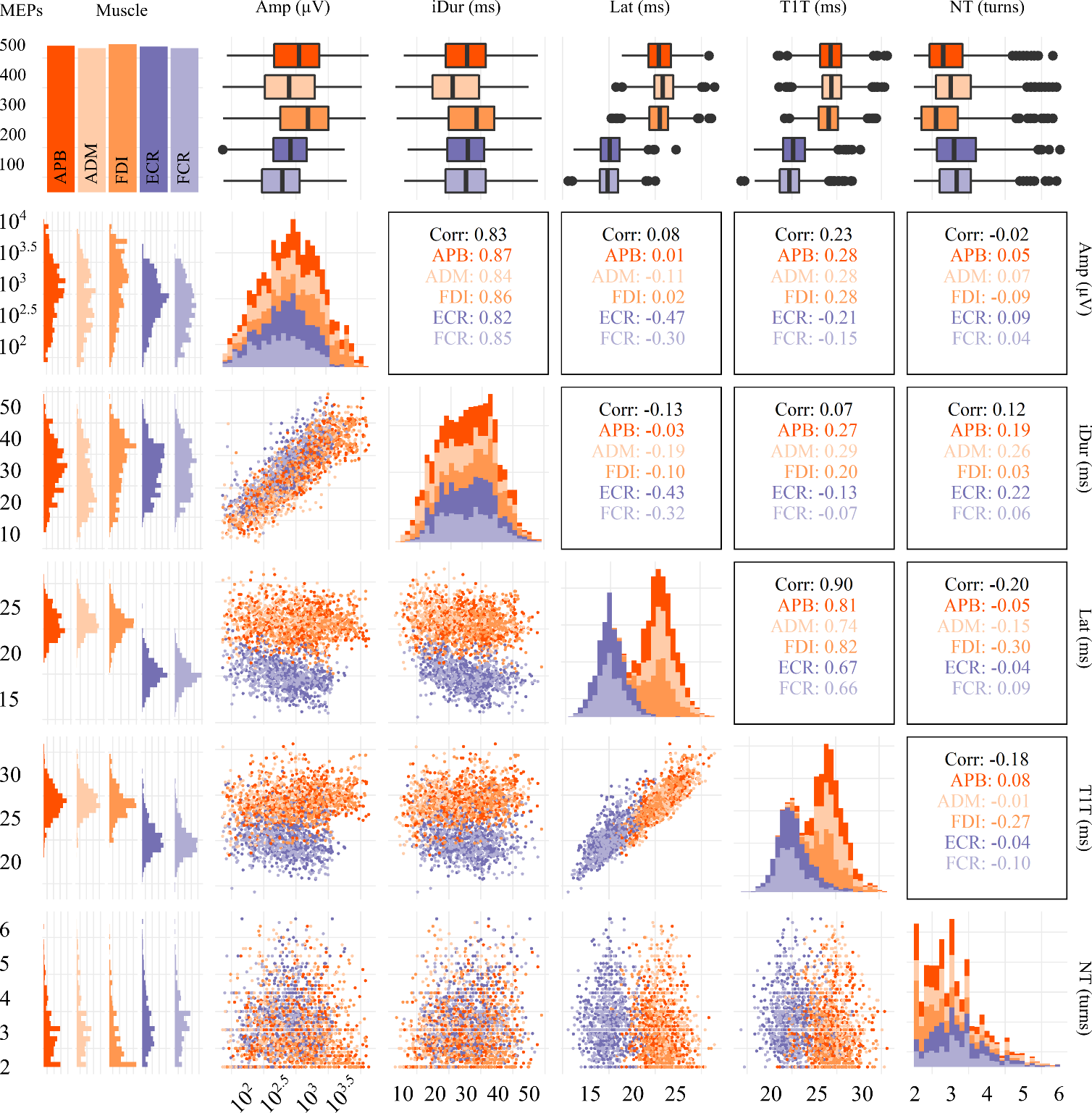


Figure S2: Feature histogram and inter-feature relationships grouped by muscles (APB: red, ADM: light orange, FDI: orange, ECR: dark purple, FCR: light purple). The box at the top-left corner depicts the sample size at each muscle, the top-most row contains boxplots of each feature, and the left-most column contains feature histograms; all are colored according to the corresponding muscle. Boxes at the diagonal show the feature histograms plotted against each muscle. Boxes above the diagonal to the right contain the correlation coefficients among features. Boxes below the diagonal to the left show scatter plots of each pair of features. Amp, amplitude; iDur, terminal-included duration; Lat, latency; T1T, the timing of the first significant turn; NT, number of turns; Cor, Correlation coefficient; MEP, motor-evoked potential; APB, abductor pollicis brevis (the primary targeted muscle); ADM, abductor digiti minimi; FDI, first dorsal interosseous; ECR, extensor carpi radialis; FCR, flexor carpi radialis.


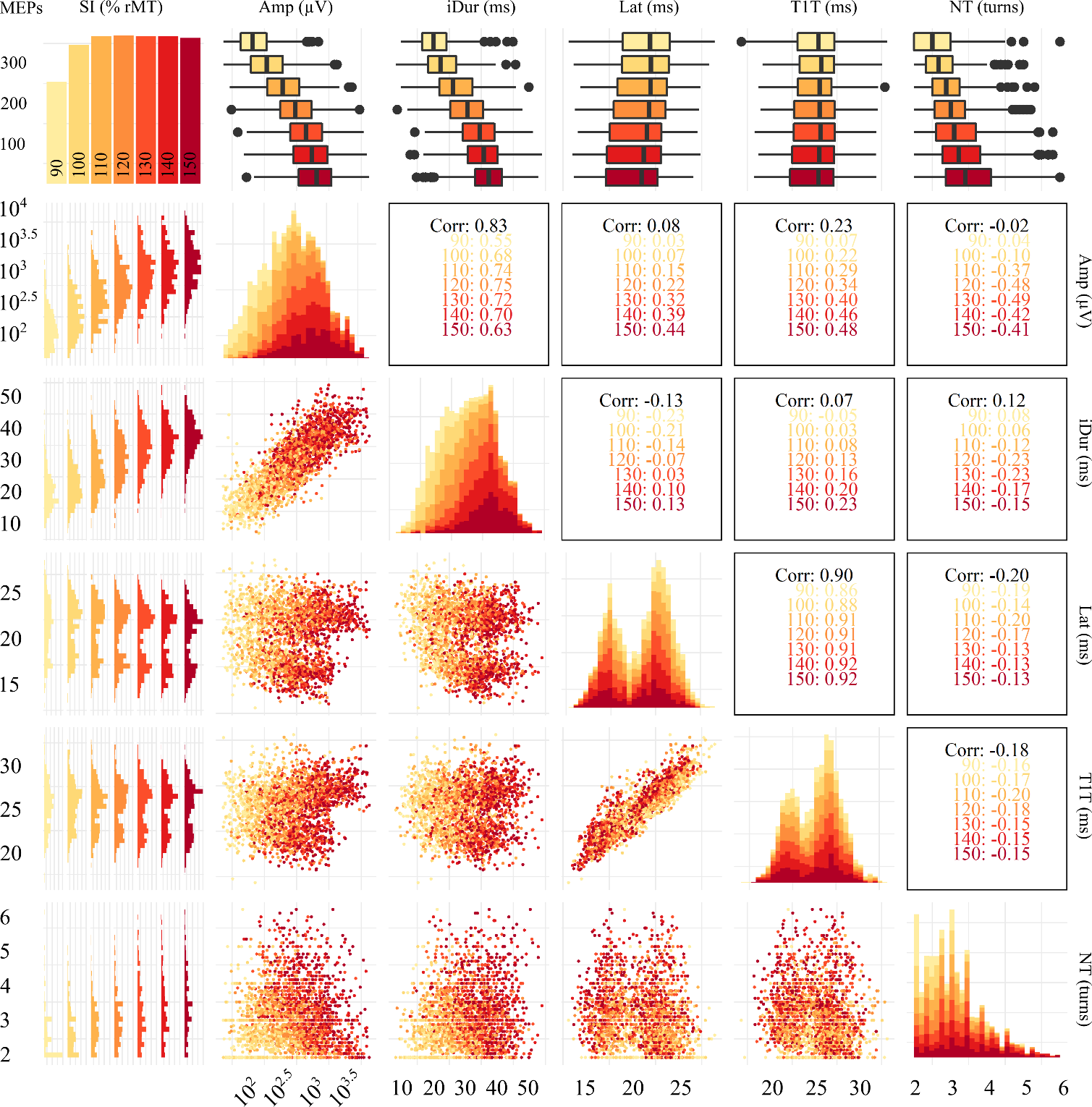


Figure S3: Feature histogram and inter-feature relationships grouped by stimulation intensity (SI) in percent of the resting motor threshold (% rMT), with the color varying lightest (90% rMT) to darkest orange (150% rMT). The box at the top-left corner depicts the sample size at each SI level, the top-most row contains boxplots of each feature, and the left-most column contains feature histograms; all are colored with the gradual increase of SI from light orange to dark orange. Along the diagonal are the feature histograms plotted in each SI level. Boxes above this diagonal to the right contain the correlation coefficients among features. Boxes below the diagonal to the left show scatter plots of each pair of features. Amp, amplitude; iDur, terminal-included duration; Lat, latency; T1T, the timing of the first significant turn; NT, number of turns; Cor, Correlation coefficient; MEP, motor-evoked potential; SI, Stimulation intensity; rMT, resting motor threshold.


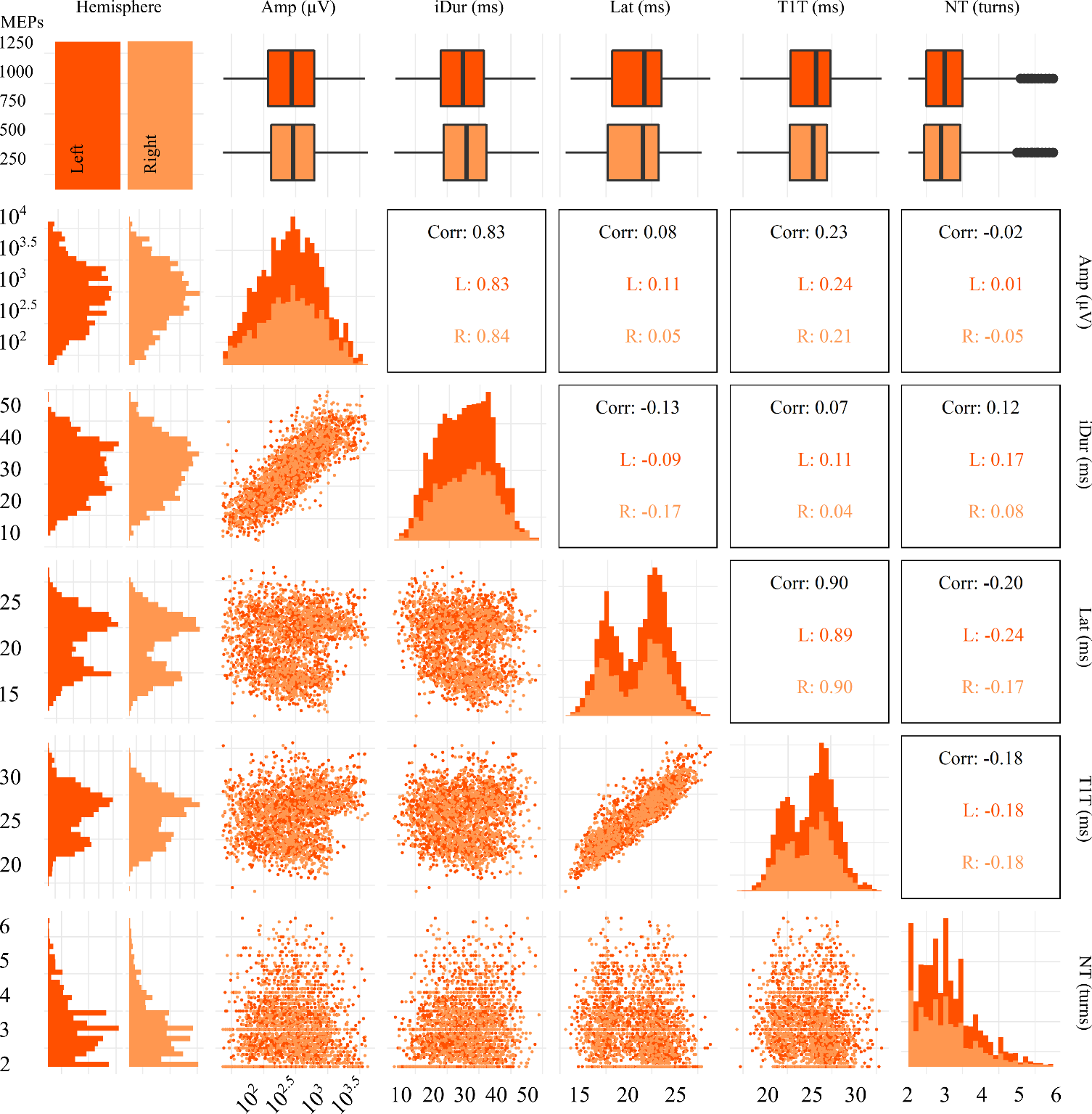


Figure S4: Feature histogram and inter-feature relationships grouped by hemisphere (Left: dark orange, Right: light orange). The box at the top-left corner depicts the sample size at each hemisphere, the top-most row contains boxplots of each feature, and the left-most column contains feature histograms; all are colored by hemisphere accordingly. Along the diagonal are the feature histograms plotted in each hemisphere. Boxes above this diagonal to the right contain the correlation coefficients among features. Boxes below the diagonal to the left show scatter plots of each pair of features. Amp, amplitude; iDur, terminal-included duration; Lat, latency; T1T, the timing of the first significant turn; NT, number of turns; Cor, Correlation coefficient; MEP, motor-evoked potential; L, left: R, right.


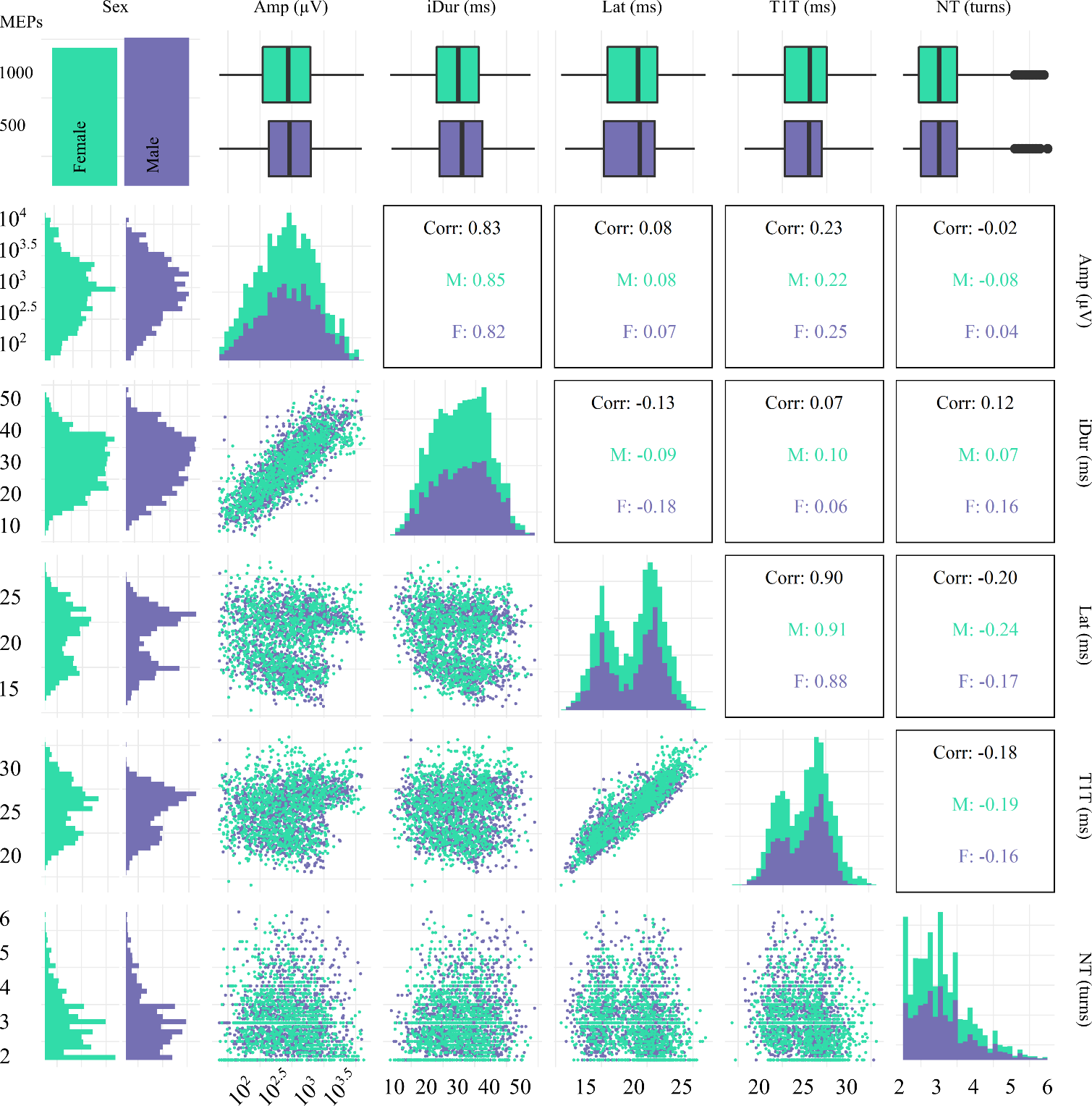


Figure S5: Feature histogram and inter-feature relationships grouped by sex (Left: dark orange, Right: light orange). The box at the top-left corner depicts the sample size at each hemisphere, the top-most row contains boxplots of each feature, and the left-most column contains feature histograms; all are colored by sex accordingly. Along the diagonal are the feature histograms plotted in each hemisphere. Boxes above this diagonal to the right contain the correlation coefficients among features. Boxes below the diagonal to the left show scatter plots of each pair of features. Amp, amplitude; iDur, terminal-included duration; Lat, latency; T1T, the timing of the first significant turn; NT, number of turns; Cor, Correlation coefficient; MEP, motor-evoked potential; M, male: F, female.

Table S1: Report table on Cramér's V from the leave-one-out cross-validation (LOOCV) test. This table shows Cramér's V of the mixed models applied on all samples (reported in Figure 3 in the manuscript), and its 95% confidence intervals (CIs) obtained by LOOCV test. **Bolded values** are outside of the 95% CIs.

| Muscle | Feature | Cramér's V [Lower boundary, Upper boundary] | | | | | |
| --- | --- | --- | --- | --- | --- | --- | --- |
|  |  | Age | SI | Hemis | Age:Hemis | Age:SI | SI:Hemis |
| APB | Amp | **0.370 [0.362, 0.367]** | **2.058 [2.021, 2.035]** | 0.073 [0.046, 0.103] | 0.265 [0.258, 0.279] | 0.191 [0.189, 0.193] | 0.131 [0.128, 0.134] |
|  | iDur | **0.257 [0.251, 0.256]** | **1.732 [1.698, 1.715]** | 0.160 [0.141, 0.181] | 0.188 [0.183, 0.199] | 0.194 [0.189, 0.196] | 0.113 [0.112, 0.117] |
|  | Lat | **0.743 [0.719, 0.737]** | **0.528 [0.515, 0.525]** | 0.433 [0.403, 0.435] | 0.082 [0.080, 0.094] | 0.176 [0.172, 0.176] | 0.254 [0.244, 0.256] |
|  | NT | 0.188 [0.184, 0.190] | 0.748 [0.729, 0.746] | 0.124 [0.113, 0.142] | 0.153 [0.145, 0.164] | 0.157 [0.154, 0.159] | 0.109 [0.105, 0.112] |
|  | T1T | **0.464 [0.448, 0.462]** | **0.349 [0.336, 0.346]** | 0.797 [0.761, 0.806] | 0.151 [0.148, 0.156] | 0.161 [0.158, 0.162] | 0.160 [0.155, 0.164] |
| ADM | Amp | **0.613 [0.597, 0.608]** | **2.199 [2.161, 2.181]** | 0.263 [0.244, 0.292] | 0.391 [0.375, 0.401] | 0.321 [0.316, 0.321] | 0.133 [0.125, 0.134] |
|  | iDur | 0.289 [0.281, 0.291] | **1.826 [1.790, 1.807]** | 0.094 [0.068, 0.109] | 0.303 [0.295, 0.307] | 0.256 [0.253, 0.257] | 0.062 [0.060, 0.071] |
|  | Lat | **0.496 [0.483, 0.494]** | **0.802 [0.783, 0.798]** | 0.050 [0.040, 0.076] | 0.378 [0.372, 0.381] | 0.117 [0.115, 0.119] | 0.109 [0.106, 0.112] |
|  | NT | 0.263 [0.258, 0.263] | **0.821 [0.806, 0.816]** | 0.008 [0.017, 0.044] | 0.437 [0.427, 0.438] | 0.203 [0.199, 0.203] | 0.139 [0.138, 0.143] |
|  | T1T | 0.485 [0.470, 0.483] | 0.168 [0.163, 0.171] | 0.550 [0.530, 0.553] | 0.167 [0.163, 0.173] | 0.162 [0.159, 0.163] | 0.143 [0.139, 0.148] |
| FDI | Amp | **0.791 [0.777, 0.787]** | **2.248 [2.206, 2.232]** | 0.150 [0.134, 0.166] | 0.173 [0.169, 0.178] | 0.285 [0.278, 0.285] | 0.122 [0.115, 0.129] |
|  | iDur | **0.620 [0.611, 0.618]** | **1.757 [1.728, 1.743]** | 0.546 [0.521, 0.557] | 0.185 [0.183, 0.186] | 0.200 [0.197, 0.200] | 0.176 [0.170, 0.177] |
|  | Lat | **0.631 [0.616, 0.626]** | **0.850 [0.835, 0.845]** | 1.118 [1.069, 1.126] | 0.196 [0.189, 0.204] | 0.178 [0.173, 0.178] | 0.101 [0.096, 0.105] |
|  | NT | **0.483 [0.473, 0.478]** | **0.882 [0.868, 0.881]** | 0.087 [0.076, 0.096] | 0.203 [0.200, 0.209] | 0.244 [0.242, 0.246] | **0.219 [0.214, 0.218]** |
|  | T1T | **0.597 [0.580, 0.593]** | 0.174 [0.171, 0.176] | 0.834 [0.795, 0.838] | 0.196 [0.188, 0.214] | 0.205 [0.202, 0.205] | 0.096 [0.094, 0.098] |
| ECR | Amp | 0.181 [0.178, 0.183] | **1.788 [1.752, 1.774]** | 0.214 [0.190, 0.235] | 0.330 [0.328, 0.340] | 0.196 [0.191, 0.196] | 0.209 [0.203, 0.212] |
|  | iDur | 0.226 [0.221, 0.230] | 1.787 [1.754, 1.770] | 0.501 [0.463, 0.510] | 0.241 [0.233, 0.248] | 0.197 [0.193, 0.197] | 0.175 [0.170, 0.177] |
|  | Lat | **0.638 [0.623, 0.634]** | **0.957 [0.938, 0.947]** | 0.635 [0.602, 0.643] | 0.295 [0.291, 0.301] | 0.129 [0.127, 0.134] | 0.137 [0.133, 0.140] |
|  | NT | 0.196 [0.190, 0.200] | **0.917 [0.894, 0.909]** | 0.068 [0.059, 0.087] | 0.265 [0.259, 0.269] | 0.164 [0.162, 0.167] | 0.128 [0.123, 0.130] |
|  | T1T | 0.210 [0.201, 0.211] | 0.449 [0.435, 0.450] | 0.295 [0.276, 0.302] | 0.294 [0.292, 0.305] | 0.081 [0.081, 0.086] | 0.077 [0.076, 0.081] |
| FCR | Amp | **0.327 [0.321, 0.327]** | **1.778 [1.748, 1.763]** | **0.484 [0.439, 0.472]** | 0.456 [0.436, 0.462] | **0.257 [0.252, 0.256]** | 0.161 [0.157, 0.164] |
|  | iDur | 0.297 [0.292, 0.299] | **1.459 [1.429, 1.447]** | 0.697 [0.658, 0.705] | 0.464 [0.454, 0.466] | 0.216 [0.213, 0.217] | 0.166 [0.162, 0.171] |
|  | Lat | **0.575 [0.558, 0.570]** | **0.950 [0.932, 0.939]** | **1.068 [1.023, 1.062]** | 0.405 [0.391, 0.408] | 0.107 [0.105, 0.110] | 0.140 [0.135, 0.143] |
|  | NT | **0.475 [0.462, 0.470]** | **0.633 [0.619, 0.629]** | 0.569 [0.537, 0.577] | 0.091 [0.091, 0.112] | 0.169 [0.168, 0.171] | 0.046 [0.045, 0.052] |
|  | T1T | 0.199 [0.194, 0.200] | **0.480 [0.467, 0.476]** | 0.400 [0.376, 0.409] | 0.382 [0.379, 0.389] | 0.180 [0.178, 0.182] | 0.122 [0.120, 0.125] |

Table S2: Report table on *p*-value from the leave-one-out cross-validation (LOOCV) test. This table shows *p*-values of the mixed models applied on all samples (reported in Figure 4 in the manuscript), and its 95% confidence intervals (CIs) obtained by LOOCV test. **Bolded values** are outside of the 95% CIs.

| Muscle | Feature | *p*-value [Lower boundary, Upper boundary] | | | | | |
| --- | --- | --- | --- | --- | --- | --- | --- |
|  |  | Age | SI | Hemis | Age:Hemis | Age:SI | SI:Hemis |
| APB | Amp | **.001 [.002, .002]** | < .0001 | .653 [.527, .753] | .046 [.031, .055] | .124 [.113, .142] | .690 [.665, .707] |
|  | iDur | **.057 [.059, .064]** | < .0001 | .323 [.267, .385] | .259 [.209, .282] | .104 [.086, .129] | .819 [.791, .828] |
|  | Lat | < .0001 | < .0001 | .008 [.007, .013] | .857 [.801, .864] | .275 [.261, .314] | .023 [.020, .035] |
|  | NT | .260 [.250, .278] | < .0001 | .443 [.381, .485] | .443 [.379, .493] | .530 [.501, .571] | .845 [.824, .865] |
|  | T1T | < .0001 | < .0001 | < .0001 | .456 [.412, .477] | .470 [.464, .511] | .439 [.410, .485] |
| ADM | Amp | < .0001 | < .0001 | .105 [.072, .133] | .001 [.000, .001] | < .0001 | .676 [.661, .737] |
|  | iDur | .023 [.022, .029] | < .0001 | .563 [.488, .667] | .015 [.013, .019] | .000 [.000, .001] | .990 [.980, .991] |
|  | Lat | < .0001 | < .0001 | .760 [.630, .803] | .001 [.001, .001] | .953 [.943, .959] | .843 [.827, .861] |
|  | NT | .049 [.048, .054] | < .0001 | **.961 [.786, .917]** | < .0001 | .061 [.058, .078] | .622 [.581, .632] |
|  | T1T | < .0001 | .378 [.352, .427] | .001 [.001, .001] | .367 [.330, .386] | .458 [.439, .509] | .586 [.535, .622] |
| FDI | Amp | < .0001 | < .0001 | .356 [.305, .406] | .332 [.311, .354] | < .0001 | .756 [.699, .804] |
|  | iDur | < .0001 | < .0001 | .001 [.001, .001] | .274 [.267, .283] | .073 [.071, .089] | .318 [.308, .358] |
|  | Lat | < .0001 | < .0001 | < .0001 | .224 [.190, .256] | .251 [.244, .305] | .887 [.866, .906] |
|  | NT | < .0001 | < .0001 | .594 [.553, .655] | .195 [.173, .206] | .002 [.001, .002] | .090 [.092, .104] |
|  | T1T | < .0001 | .327 [.313, .353] | < .0001 | .223 [.151, .259] | .051 [.048, .065] | .909 [.903, .919] |
| ECR | Amp | .293 [.279, .309] | < .0001 | .187 [.148, .242] | .006 [.004, .006] | .094 [.089, .125] | .126 [.114, .154] |
|  | iDur | .119 [.108, .135] | < .0001 | .002 [.002, .004] | .085 [.068, .104] | .088 [.085, .114] | .323 [.303, .350] |
|  | Lat | < .0001 | < .0001 | < .0001 | .019 [.016, .022] | .874 [.834, .891] | .638 [.618, .671] |
|  | NT | .224 [.209, .251] | < .0001 | .674 [.592, .716] | .046 [.039, .053] | .428 [.394, .469] | .715 [.696, .746] |
|  | T1T | .170 [.166, .201] | < .0001 | .069 [.062, .089] | .020 [.014, .021] | .999 [.999, .999] | .970 [.959, .970] |
| FCR | Amp | .007 [.007, .008] | < .0001 | .003 [.003, .006] | < .0001 | .000 [.000, .001] | .433 [.405, .466] |
|  | iDur | .018 [.017, .021] | < .0001 | < .0001 | < .0001 | .022 [.021, .028] | .391 [.348, .425] |
|  | Lat | < .0001 | < .0001 | < .0001 | .000 [.000, .001] | .982 [.974, .984] | .610 [.586, .657] |
|  | NT | < .0001 | < .0001 | .000 [.000, .001] | .816 [.698, .816] | .362 [.335, .374] | .998 [.996, .998] |
|  | T1T | .212 [.209, .231] | < .0001 | .014 [.011, .021] | .001 [.001, .001] | .223 [.207, .242] | .757 [.735, .770] |

Table S3: Statistics Summary of linear mixed models fitting

===========================================================================

Investigating feature: Amp of APB

===========================================================================

Linear mixed model fit by maximum likelihood . t-tests use Satterthwaite's method ['lmerModLmerTest']

Formula: curFormula

Data: TMSdev

AIC BIC logLik deviance df.resid

158.8 326.0 -39.4 78.8 443

Scaled residuals:

Min 1Q Median 3Q Max

-2.7028 -0.5944 0.0333 0.6065 3.7131

Random effects:

Groups Name Variance Std.Dev.

ID (Intercept) 0.03925 0.1981

Residual 0.05771 0.2402

Number of obs: 483, groups: ID, 38

Fixed effects:

Estimate Std. Error df t value Pr(>|t|)

(Intercept) 2.066132 0.110822 134.630217 18.644 < 2e-16 ***

AgepAdolesc 0.071898 0.143047 129.972584 0.503 0.616084

AgeAdolesc 0.218781 0.142470 136.554261 1.536 0.126944

AgeAdlt 0.001416 0.141701 125.649475 0.010 0.992043

SI100 0.131146 0.108056 445.461231 1.214 0.225511

SI110 0.304201 0.106751 445.531938 2.850 0.004580 **

SI120 0.509287 0.106719 445.551215 4.772 2.47e-06 ***

SI130 0.612351 0.106670 445.533721 5.741 1.75e-08 ***

SI140 0.703811 0.106718 445.546238 6.595 1.21e-10 ***

SI150 0.817972 0.106718 445.546238 7.665 1.13e-13 ***

HemisR -0.029317 0.083110 451.144540 -0.353 0.724437

AgepAdolesc:HemisR 0.168510 0.067834 451.500950 2.484 0.013347 *

AgeAdolesc:HemisR 0.047313 0.067620 451.788708 0.700 0.484483

AgeAdlt:HemisR 0.039500 0.068670 453.960210 0.575 0.565432

AgepAdolesc:SI100 0.111741 0.133887 445.874565 0.835 0.404394

AgeAdolesc:SI100 0.018642 0.135238 446.228220 0.138 0.890422

AgeAdlt:SI100 0.268656 0.131016 445.330877 2.051 0.040894 *

AgepAdolesc:SI110 0.127919 0.131133 445.791849 0.975 0.329844

AgeAdolesc:SI110 0.090631 0.133146 447.123329 0.681 0.496418

AgeAdlt:SI110 0.371467 0.129536 445.472129 2.868 0.004331 **

AgepAdolesc:SI120 0.084902 0.131132 445.791996 0.647 0.517673

AgeAdolesc:SI120 0.067032 0.132304 446.577146 0.507 0.612649

AgeAdlt:SI120 0.404725 0.129535 445.472587 3.124 0.001898 **

AgepAdolesc:SI130 0.241309 0.131132 445.791837 1.840 0.066403 .

AgeAdolesc:SI130 0.177581 0.131676 446.221933 1.349 0.178145

AgeAdlt:SI130 0.491707 0.129534 445.472096 3.796 0.000167 ***

AgepAdolesc:SI140 0.250550 0.131132 445.791965 1.911 0.056689 .

AgeAdolesc:SI140 0.198363 0.132275 446.472769 1.500 0.134418

AgeAdlt:SI140 0.497387 0.129535 445.472474 3.840 0.000141 ***

AgepAdolesc:SI150 0.176580 0.131132 445.791965 1.347 0.178800

AgeAdolesc:SI150 0.159707 0.132275 446.472769 1.207 0.227922

AgeAdlt:SI150 0.431980 0.129535 445.472474 3.335 0.000925 ***

SI100:HemisR -0.145921 0.090648 446.311135 -1.610 0.108161

SI110:HemisR -0.027368 0.089228 446.436062 -0.307 0.759200

SI120:HemisR -0.009202 0.089049 446.567816 -0.103 0.917743

SI130:HemisR -0.061548 0.088770 446.455313 -0.693 0.488454

SI140:HemisR -0.053539 0.089043 446.535844 -0.601 0.547964

SI150:HemisR -0.054386 0.089043 446.535844 -0.611 0.541649

---

Signif. codes: 0 ‘***’ 0.001 ‘**’ 0.01 ‘*’ 0.05 ‘.’ 0.1 ‘ ’ 1

===========================================================================

Investigating feature: iDur of APB

===========================================================================

Linear mixed model fit by maximum likelihood . t-tests use Satterthwaite's method ['lmerModLmerTest']

Formula: curFormula

Data: TMSdev

AIC BIC logLik deviance df.resid

3070.2 3237.4 -1495.1 2990.2 443

Scaled residuals:

Min 1Q Median 3Q Max

-2.7652 -0.6139 0.0281 0.6338 3.4174

Random effects:

Groups Name Variance Std.Dev.

ID (Intercept) 11.33 3.366

Residual 24.58 4.958

Number of obs: 483, groups: ID, 38

Fixed effects:

Estimate Std. Error df t value Pr(>|t|)

(Intercept) 19.9120 2.1121 180.3192 9.428 < 2e-16 ***

AgepAdolesc 0.1245 2.7213 173.5803 0.046 0.96356

AgeAdolesc 1.9593 2.7170 182.5921 0.721 0.47176

AgeAdlt -2.5104 2.6909 167.2507 -0.933 0.35221

SI100 2.4071 2.2300 445.7060 1.079 0.28099

SI110 5.2784 2.2030 445.8063 2.396 0.01699 *

SI120 7.3409 2.2024 445.8327 3.333 0.00093 ***

SI130 9.5976 2.2014 445.8088 4.360 1.62e-05 ***

SI140 11.8854 2.2024 445.8262 5.397 1.10e-07 ***

SI150 14.2058 2.2024 445.8262 6.450 2.91e-10 ***

HemisR 0.1693 1.7129 453.2305 0.099 0.92133

AgepAdolesc:HemisR 2.3145 1.3980 453.5878 1.656 0.09850 .

AgeAdolesc:HemisR 0.2238 1.3935 453.9777 0.161 0.87247

AgeAdlt:HemisR 1.2616 1.4144 456.7492 0.892 0.37286

AgepAdolesc:SI100 0.8110 2.7628 446.2830 0.294 0.76925

AgeAdolesc:SI100 0.2357 2.7905 446.7594 0.084 0.93274

AgeAdlt:SI100 2.6933 2.7039 445.5269 0.996 0.31975

AgepAdolesc:SI110 2.4260 2.7060 446.1722 0.897 0.37047

AgeAdolesc:SI110 0.5120 2.7468 447.9837 0.186 0.85222

AgeAdlt:SI110 4.8734 2.6733 445.7248 1.823 0.06897 .

AgepAdolesc:SI120 0.6589 2.7060 446.1724 0.243 0.80775

AgeAdolesc:SI120 1.9598 2.7297 447.2399 0.718 0.47317

AgeAdlt:SI120 6.4103 2.6733 445.7254 2.398 0.01690 *

AgepAdolesc:SI130 3.5096 2.7060 446.1722 1.297 0.19532

AgeAdolesc:SI130 5.3655 2.7170 446.7559 1.975 0.04891 *

AgeAdlt:SI130 8.6151 2.6733 445.7248 3.223 0.00136 **

AgepAdolesc:SI140 3.2279 2.7060 446.1724 1.193 0.23356

AgeAdolesc:SI140 5.9487 2.7292 447.1042 2.180 0.02980 *

AgeAdlt:SI140 7.4795 2.6733 445.7253 2.798 0.00537 **

AgepAdolesc:SI150 1.7356 2.7060 446.1724 0.641 0.52161

AgeAdolesc:SI150 4.6788 2.7292 447.1042 1.714 0.08715 .

AgeAdlt:SI150 5.9439 2.6733 445.7253 2.223 0.02668 *

SI100:HemisR -2.0398 1.8704 446.8909 -1.091 0.27605

SI110:HemisR -0.7925 1.8410 447.0687 -0.430 0.66707

SI120:HemisR 0.5587 1.8373 447.2487 0.304 0.76118

SI130:HemisR -0.9790 1.8316 447.0956 -0.535 0.59323

SI140:HemisR -1.2695 1.8372 447.2072 -0.691 0.48993

SI150:HemisR -0.6385 1.8372 447.2072 -0.348 0.72836

---

Signif. codes: 0 ‘***’ 0.001 ‘**’ 0.01 ‘*’ 0.05 ‘.’ 0.1 ‘ ’ 1

===========================================================================

Investigating feature: Lat of APB

===========================================================================

Linear mixed model fit by maximum likelihood . t-tests use Satterthwaite's method ['lmerModLmerTest']

Formula: curFormula

Data: TMSdev

AIC BIC logLik deviance df.resid

1347.5 1514.7 -633.7 1267.5 443

Scaled residuals:

Min 1Q Median 3Q Max

-3.3907 -0.5540 -0.0218 0.5520 3.5642

Random effects:

Groups Name Variance Std.Dev.

ID (Intercept) 0.6928 0.8324

Residual 0.6550 0.8093

Number of obs: 483, groups: ID, 38

Fixed effects:

Estimate Std. Error df t value Pr(>|t|)

(Intercept) 23.3236 0.4180 97.9116 55.792 < 2e-16 ***

AgepAdolesc -0.7141 0.5407 95.0505 -1.321 0.189809

AgeAdolesc 1.9053 0.5370 99.2499 3.548 0.000594 ***

AgeAdlt 1.1404 0.5367 92.4195 2.125 0.036268 *

SI100 -1.0124 0.3641 445.0052 -2.781 0.005654 **

SI110 -1.2922 0.3597 445.0523 -3.593 0.000364 ***

SI120 -1.5790 0.3596 445.0655 -4.391 1.41e-05 ***

SI130 -1.7011 0.3594 445.0535 -4.733 2.98e-06 ***

SI140 -2.2517 0.3596 445.0620 -6.262 8.96e-10 ***

SI150 -2.2464 0.3596 445.0620 -6.247 9.77e-10 ***

HemisR -0.8386 0.2803 449.0246 -2.991 0.002929 **

AgepAdolesc:HemisR -0.1906 0.2288 449.3278 -0.833 0.405315

AgeAdolesc:HemisR -0.1616 0.2281 449.5274 -0.708 0.479180

AgeAdlt:HemisR -0.1138 0.2318 451.1102 -0.491 0.623614

AgepAdolesc:SI100 0.9042 0.4512 445.2845 2.004 0.045665 *

AgeAdolesc:SI100 0.3241 0.4557 445.5306 0.711 0.477418

AgeAdlt:SI100 0.5885 0.4414 444.9158 1.333 0.183200

AgepAdolesc:SI110 0.6735 0.4419 445.2267 1.524 0.128187

AgeAdolesc:SI110 0.5152 0.4488 446.1459 1.148 0.251539

AgeAdlt:SI110 0.5088 0.4365 445.0110 1.166 0.244367

AgepAdolesc:SI120 1.1711 0.4419 445.2268 2.650 0.008329 **

AgeAdolesc:SI120 0.8581 0.4459 445.7691 1.925 0.054925 .

AgeAdlt:SI120 0.7180 0.4365 445.0113 1.645 0.100662

AgepAdolesc:SI130 0.8924 0.4419 445.2267 2.020 0.044025 *

AgeAdolesc:SI130 0.9931 0.4437 445.5242 2.238 0.025713 *

AgeAdlt:SI130 0.5682 0.4365 445.0110 1.302 0.193618

AgepAdolesc:SI140 1.1063 0.4419 445.2268 2.504 0.012644 *

AgeAdolesc:SI140 1.2451 0.4458 445.6944 2.793 0.005446 **

AgeAdlt:SI140 1.1760 0.4365 445.0113 2.694 0.007316 **

AgepAdolesc:SI150 1.1445 0.4419 445.2268 2.590 0.009912 **

AgeAdolesc:SI150 1.0844 0.4458 445.6944 2.433 0.015379 *

AgeAdlt:SI150 0.9028 0.4365 445.0113 2.068 0.039185 *

SI100:HemisR 0.5808 0.3055 445.5802 1.901 0.057930 .

SI110:HemisR 0.9258 0.3007 445.6632 3.079 0.002207 **

SI120:HemisR 0.7970 0.3001 445.7539 2.656 0.008194 **

SI130:HemisR 0.9267 0.2992 445.6762 3.098 0.002073 **

SI140:HemisR 0.9330 0.3001 445.7310 3.109 0.001997 **

SI150:HemisR 0.9332 0.3001 445.7310 3.110 0.001992 **

---

Signif. codes: 0 ‘***’ 0.001 ‘**’ 0.01 ‘*’ 0.05 ‘.’ 0.1 ‘ ’ 1

===========================================================================

Investigating feature: T1T of APB

===========================================================================

Linear mixed model fit by maximum likelihood . t-tests use Satterthwaite's method ['lmerModLmerTest']

Formula: curFormula

Data: TMSdev

AIC BIC logLik deviance df.resid

1369.4 1536.6 -644.7 1289.4 443

Scaled residuals:

Min 1Q Median 3Q Max

-4.0849 -0.5051 -0.0162 0.5312 3.8648

Random effects:

Groups Name Variance Std.Dev.

ID (Intercept) 1.3438 1.1592

Residual 0.6522 0.8076

Number of obs: 483, groups: ID, 38

Fixed effects:

Estimate Std. Error df t value Pr(>|t|)

(Intercept) 26.5087 0.5172 67.3702 51.257 < 2e-16 ***

AgepAdolesc -0.9905 0.6708 66.0365 -1.477 0.14456

AgeAdolesc 1.6439 0.6634 68.0518 2.478 0.01570 *

AgeAdlt 0.7776 0.6676 64.8182 1.165 0.24838

SI100 -0.4946 0.3634 445.0493 -1.361 0.17411

SI110 -0.9026 0.3590 445.0740 -2.514 0.01227 *

SI120 -0.5219 0.3589 445.0811 -1.454 0.14654

SI130 -0.5684 0.3587 445.0746 -1.585 0.11374

SI140 -0.6650 0.3589 445.0791 -1.853 0.06454 .

SI150 -0.5668 0.3589 445.0791 -1.579 0.11497

HemisR -0.5775 0.2801 447.2810 -2.062 0.03980 *

AgepAdolesc:HemisR -0.1348 0.2286 447.4767 -0.590 0.55576

AgeAdolesc:HemisR -0.1922 0.2279 447.5853 -0.843 0.39946

AgeAdlt:HemisR -0.3593 0.2317 448.4893 -1.551 0.12171

AgepAdolesc:SI100 0.8087 0.4503 445.1976 1.796 0.07317 .

AgeAdolesc:SI100 0.2576 0.4549 445.3319 0.566 0.57149

AgeAdlt:SI100 0.5223 0.4405 445.0012 1.186 0.23638

AgepAdolesc:SI110 0.9556 0.4410 445.1659 2.167 0.03078 *

AgeAdolesc:SI110 0.5314 0.4480 445.6642 1.186 0.23616

AgeAdlt:SI110 1.0239 0.4356 445.0516 2.351 0.01918 *

AgepAdolesc:SI120 0.8873 0.4410 445.1660 2.012 0.04482 *

AgeAdolesc:SI120 0.5039 0.4451 445.4601 1.132 0.25815

AgeAdlt:SI120 0.8415 0.4356 445.0518 1.932 0.05399 .

AgepAdolesc:SI130 1.2466 0.4410 445.1659 2.827 0.00491 **

AgeAdolesc:SI130 0.9378 0.4429 445.3274 2.117 0.03478 *

AgeAdlt:SI130 0.9410 0.4356 445.0516 2.160 0.03128 *

AgepAdolesc:SI140 1.2425 0.4410 445.1660 2.818 0.00505 **

AgeAdolesc:SI140 0.8855 0.4450 445.4183 1.990 0.04719 *

AgeAdlt:SI140 0.9374 0.4356 445.0517 2.152 0.03192 *

AgepAdolesc:SI150 1.3762 0.4410 445.1660 3.121 0.00192 **

AgeAdolesc:SI150 0.8148 0.4450 445.4183 1.831 0.06774 .

AgeAdlt:SI150 0.9670 0.4356 445.0517 2.220 0.02692 *

SI100:HemisR 0.2107 0.3049 445.3550 0.691 0.48982

SI110:HemisR 0.5960 0.3001 445.3984 1.986 0.04770 *

SI120:HemisR 0.5211 0.2996 445.4473 1.740 0.08260 .

SI130:HemisR 0.4286 0.2986 445.4053 1.435 0.15193

SI140:HemisR 0.5228 0.2995 445.4345 1.745 0.08164 .

SI150:HemisR 0.3449 0.2995 445.4345 1.152 0.25014

---

Signif. codes: 0 ‘***’ 0.001 ‘**’ 0.01 ‘*’ 0.05 ‘.’ 0.1 ‘ ’ 1

===========================================================================

Investigating feature: NT of APB

===========================================================================

Linear mixed model fit by maximum likelihood . t-tests use Satterthwaite's method ['lmerModLmerTest']

Formula: curFormula

Data: TMSdev

AIC BIC logLik deviance df.resid

886.9 1054.1 -403.4 806.9 443

Scaled residuals:

Min 1Q Median 3Q Max

-2.9559 -0.6690 -0.0675 0.5957 3.7713

Random effects:

Groups Name Variance Std.Dev.

ID (Intercept) 0.1616 0.4019

Residual 0.2624 0.5122

Number of obs: 483, groups: ID, 38

Fixed effects:

Estimate Std. Error df t value Pr(>|t|)

(Intercept) 2.967018 0.231128 145.305105 12.837 <2e-16 ***

AgepAdolesc -0.491557 0.298194 140.145920 -1.648 0.1015

AgeAdolesc -0.479944 0.297187 147.350874 -1.615 0.1085

AgeAdlt -0.473197 0.295252 135.343909 -1.603 0.1113

SI100 0.129292 0.230396 445.610966 0.561 0.5750

SI110 0.035160 0.227613 445.688214 0.154 0.8773

SI120 0.130901 0.227545 445.709107 0.575 0.5654

SI130 0.209271 0.227440 445.690158 0.920 0.3580

SI140 0.415712 0.227542 445.703772 1.827 0.0684 .

SI150 0.581462 0.227542 445.703772 2.555 0.0109 *

HemisR -0.125726 0.177152 451.721072 -0.710 0.4783

AgepAdolesc:HemisR -0.178237 0.144587 452.082788 -1.233 0.2183

AgeAdolesc:HemisR -0.157824 0.144129 452.393810 -1.095 0.2741

AgeAdlt:HemisR -0.021167 0.146350 454.708749 -0.145 0.8851

AgepAdolesc:SI100 -0.003618 0.285467 446.060882 -0.013 0.9899

AgeAdolesc:SI100 -0.163018 0.288342 446.442694 -0.565 0.5721

AgeAdlt:SI100 -0.002198 0.279352 445.469607 -0.008 0.9937

AgepAdolesc:SI110 0.273115 0.279595 445.971683 0.977 0.3292

AgeAdolesc:SI110 0.170883 0.283867 447.412404 0.602 0.5475

AgeAdlt:SI110 0.228207 0.276195 445.623485 0.826 0.4091

AgepAdolesc:SI120 0.317927 0.279594 445.971843 1.137 0.2561

AgeAdolesc:SI120 0.305240 0.282080 446.821285 1.082 0.2798

AgeAdlt:SI120 0.185153 0.276193 445.623981 0.670 0.5030

AgepAdolesc:SI130 0.175291 0.279594 445.971670 0.627 0.5310

AgeAdolesc:SI130 0.532926 0.280748 446.436801 1.898 0.0583 .

AgeAdlt:SI130 0.206503 0.276191 445.623449 0.748 0.4550

AgepAdolesc:SI140 0.243328 0.279594 445.971810 0.870 0.3846

AgeAdolesc:SI140 0.478764 0.282020 446.709494 1.698 0.0903 .

AgeAdlt:SI140 0.159512 0.276193 445.623860 0.578 0.5639

AgepAdolesc:SI150 0.116640 0.279594 445.971810 0.417 0.6768

AgeAdolesc:SI150 0.403073 0.282020 446.709494 1.429 0.1536

AgeAdlt:SI150 0.188039 0.276193 445.623860 0.681 0.4963

SI100:HemisR 0.216410 0.193271 446.535791 1.120 0.2634

SI110:HemisR 0.246493 0.190242 446.672375 1.296 0.1958

SI120:HemisR 0.272386 0.189857 446.815083 1.435 0.1521

SI130:HemisR 0.225919 0.189264 446.693331 1.194 0.2332

SI140:HemisR 0.141963 0.189845 446.780846 0.748 0.4550

SI150:HemisR 0.162839 0.189845 446.780846 0.858 0.3915

---

Signif. codes: 0 ‘***’ 0.001 ‘**’ 0.01 ‘*’ 0.05 ‘.’ 0.1 ‘ ’ 1

===========================================================================

Investigating feature: Amp of ADM

===========================================================================

Linear mixed model fit by maximum likelihood . t-tests use Satterthwaite's method ['lmerModLmerTest']

Formula: curFormula

Data: TMSdev

AIC BIC logLik deviance df.resid

84.6 250.2 -2.3 4.6 425

Scaled residuals:

Min 1Q Median 3Q Max

-2.58849 -0.70064 0.02369 0.65603 2.78104

Random effects:

Groups Name Variance Std.Dev.

ID (Intercept) 0.02329 0.1526

Residual 0.05074 0.2253

Number of obs: 465, groups: ID, 38

Fixed effects:

Estimate Std. Error df t value Pr(>|t|)

(Intercept) 1.947741 0.096917 171.796522 20.097 < 2e-16 ***

AgepAdolesc -0.006824 0.123572 158.007828 -0.055 0.956033

AgeAdolesc 0.018739 0.130245 198.643844 0.144 0.885743

AgeAdlt 0.165302 0.134131 203.828332 1.232 0.219223

SI100 0.240911 0.106503 425.666593 2.262 0.024200 *

SI110 0.261604 0.101265 424.541109 2.583 0.010118 *

SI120 0.334915 0.101322 424.411920 3.305 0.001029 **

SI130 0.484972 0.101325 424.364578 4.786 2.35e-06 ***

SI140 0.559517 0.102260 424.400566 5.472 7.64e-08 ***

SI150 0.646558 0.103767 424.816544 6.231 1.12e-09 ***

HemisR 0.053031 0.083723 432.832362 0.633 0.526801

AgepAdolesc:HemisR 0.048011 0.065436 433.410219 0.734 0.463525

AgeAdolesc:HemisR -0.152788 0.064537 433.285626 -2.367 0.018351 *

AgeAdlt:HemisR -0.141214 0.066661 435.834374 -2.118 0.034707 *

AgepAdolesc:SI100 -0.038979 0.125649 425.963857 -0.310 0.756544

AgeAdolesc:SI100 0.015976 0.133214 426.252583 0.120 0.904597

AgeAdlt:SI100 0.178468 0.134947 425.454341 1.323 0.186711

AgepAdolesc:SI110 0.116309 0.122641 425.358282 0.948 0.343478

AgeAdolesc:SI110 0.279206 0.129803 425.519598 2.151 0.032039 *

AgeAdlt:SI110 0.376532 0.131342 425.635550 2.867 0.004352 **

AgepAdolesc:SI120 0.301019 0.123130 424.657932 2.445 0.014901 *

AgeAdolesc:SI120 0.455047 0.130437 426.112801 3.489 0.000536 ***

AgeAdlt:SI120 0.525999 0.131345 425.633197 4.005 7.33e-05 ***

AgepAdolesc:SI130 0.343491 0.123916 424.954557 2.772 0.005817 **

AgeAdolesc:SI130 0.578776 0.129827 425.545838 4.458 1.06e-05 ***

AgeAdlt:SI130 0.537156 0.131344 425.629534 4.090 5.17e-05 ***

AgepAdolesc:SI140 0.334924 0.124010 425.019247 2.701 0.007194 **

AgeAdolesc:SI140 0.639012 0.130586 425.074411 4.893 1.41e-06 ***

AgeAdlt:SI140 0.599235 0.132659 425.371139 4.517 8.13e-06 ***

AgepAdolesc:SI150 0.338872 0.126588 425.686654 2.677 0.007716 **

AgeAdolesc:SI150 0.646585 0.132417 425.402441 4.883 1.48e-06 ***

AgeAdlt:SI150 0.614521 0.134572 425.694523 4.566 6.50e-06 ***

SI100:HemisR -0.117401 0.091171 426.121307 -1.288 0.198549

SI110:HemisR 0.003549 0.090159 426.844400 0.039 0.968616

SI120:HemisR -0.009175 0.090464 426.082159 -0.101 0.919264

SI130:HemisR 0.019292 0.090479 425.803132 0.213 0.831260

SI140:HemisR -0.020922 0.090144 426.263888 -0.232 0.816573

SI150:HemisR -0.005873 0.090817 426.352192 -0.065 0.948469

---

Signif. codes: 0 ‘***’ 0.001 ‘**’ 0.01 ‘*’ 0.05 ‘.’ 0.1 ‘ ’ 1

===========================================================================

Investigating feature: iDur of ADM

===========================================================================

Linear mixed model fit by maximum likelihood . t-tests use Satterthwaite's method ['lmerModLmerTest']

Formula: curFormula

Data: TMSdev

AIC BIC logLik deviance df.resid

2969.1 3134.8 -1444.5 2889.1 425

Scaled residuals:

Min 1Q Median 3Q Max

-2.5393 -0.6505 -0.0376 0.6268 4.3298

Random effects:

Groups Name Variance Std.Dev.

ID (Intercept) 13.98 3.739

Residual 24.72 4.972

Number of obs: 465, groups: ID, 38

Fixed effects:

Estimate Std. Error df t value Pr(>|t|)

(Intercept) 18.5547 2.2257 148.9239 8.337 4.70e-14 ***

AgepAdolesc -4.5363 2.8435 137.7762 -1.595 0.112936

AgeAdolesc -2.0721 2.9805 172.0232 -0.695 0.487858

AgeAdlt -2.6653 3.0675 176.7197 -0.869 0.386095

SI100 -0.3191 2.3513 426.2209 -0.136 0.892110

SI110 2.7895 2.2354 425.2379 1.248 0.212773

SI120 3.8430 2.2367 425.1276 1.718 0.086496 .

SI130 7.6749 2.2367 425.0878 3.431 0.000659 ***

SI140 9.3279 2.2574 425.1223 4.132 4.33e-05 ***

SI150 10.7693 2.2907 425.4626 4.701 3.50e-06 ***

HemisR 0.4037 1.8496 432.2787 0.218 0.827329

AgepAdolesc:HemisR 2.7383 1.4457 432.8672 1.894 0.058884 .

AgeAdolesc:HemisR -0.8286 1.4259 432.7319 -0.581 0.561444

AgeAdlt:HemisR -0.8138 1.4731 434.9209 -0.552 0.580963

AgepAdolesc:SI100 2.4898 2.7741 426.4685 0.898 0.369952

AgeAdolesc:SI100 4.2909 2.9412 426.6671 1.459 0.145326

AgeAdlt:SI100 5.4565 2.9792 426.0038 1.832 0.067723 .

AgepAdolesc:SI110 4.5650 2.7075 425.9458 1.686 0.092517 .

AgeAdolesc:SI110 3.5935 2.8657 426.0387 1.254 0.210541

AgeAdlt:SI110 6.1229 2.8997 426.1311 2.112 0.035305 *

AgepAdolesc:SI120 7.1448 2.7181 425.3267 2.629 0.008885 **

AgeAdolesc:SI120 8.7039 2.8798 426.5382 3.022 0.002659 **

AgeAdlt:SI120 10.1723 2.8997 426.1292 3.508 0.000499 ***

AgepAdolesc:SI130 8.0414 2.7356 425.5707 2.940 0.003466 **

AgeAdolesc:SI130 10.7029 2.8662 426.0608 3.734 0.000214 ***

AgeAdlt:SI130 10.0147 2.8997 426.1261 3.454 0.000608 ***

AgepAdolesc:SI140 7.3216 2.7377 425.6600 2.674 0.007774 **

AgeAdolesc:SI140 11.8619 2.8828 425.6678 4.115 4.66e-05 ***

AgeAdlt:SI140 10.9618 2.9287 425.9139 3.743 0.000207 ***

AgepAdolesc:SI150 7.5180 2.7947 426.2106 2.690 0.007425 **

AgeAdolesc:SI150 12.2940 2.9233 425.9365 4.205 3.18e-05 ***

AgeAdlt:SI150 11.9418 2.9710 426.1789 4.019 6.90e-05 ***

SI100:HemisR -0.3738 2.0129 426.5586 -0.186 0.852752

SI110:HemisR -0.9788 1.9907 427.1525 -0.492 0.623173

SI120:HemisR -1.4514 1.9973 426.5007 -0.727 0.467818

SI130:HemisR -1.2907 1.9976 426.2655 -0.646 0.518546

SI140:HemisR -1.1908 1.9902 426.6711 -0.598 0.549958

SI150:HemisR -1.0414 2.0051 426.7476 -0.519 0.603770

---

Signif. codes: 0 ‘***’ 0.001 ‘**’ 0.01 ‘*’ 0.05 ‘.’ 0.1 ‘ ’ 1

===========================================================================

Investigating feature: Lat of ADM

===========================================================================

Linear mixed model fit by maximum likelihood . t-tests use Satterthwaite's method ['lmerModLmerTest']

Formula: curFormula

Data: TMSdev

AIC BIC logLik deviance df.resid

1369.3 1534.9 -644.6 1289.3 425

Scaled residuals:

Min 1Q Median 3Q Max

-3.3441 -0.6755 0.0180 0.6081 3.1422

Random effects:

Groups Name Variance Std.Dev.

ID (Intercept) 0.7337 0.8566

Residual 0.7620 0.8729

Number of obs: 465, groups: ID, 38

Fixed effects:

Estimate Std. Error df t value Pr(>|t|)

(Intercept) 23.46597 0.44273 106.30945 53.003 < 2e-16 ***

AgepAdolesc -0.25439 0.56848 100.11329 -0.447 0.65548

AgeAdolesc 1.55299 0.58720 120.67294 2.645 0.00926 **

AgeAdlt 1.48897 0.60322 123.83463 2.468 0.01494 *

SI100 0.23119 0.41301 428.05092 0.560 0.57594

SI110 -0.33615 0.39257 427.39987 -0.856 0.39232

SI120 -0.39095 0.39278 427.32974 -0.995 0.32013

SI130 -0.85635 0.39279 427.30518 -2.180 0.02979 *

SI140 -1.18105 0.39641 427.33177 -2.979 0.00305 **

SI150 -1.23696 0.40230 427.53014 -3.075 0.00224 **

HemisR 0.05290 0.32533 431.82635 0.163 0.87091

AgepAdolesc:HemisR -0.41908 0.25433 432.32240 -1.648 0.10012

AgeAdolesc:HemisR 0.45258 0.25082 432.19593 1.804 0.07187 .

AgeAdlt:HemisR -0.09397 0.25927 433.60090 -0.362 0.71721

AgepAdolesc:SI100 -0.22274 0.48729 428.20425 -0.457 0.64784

AgeAdolesc:SI100 -0.63184 0.51666 428.26834 -1.223 0.22203

AgeAdlt:SI100 -0.51057 0.52327 427.86944 -0.976 0.32976

AgepAdolesc:SI110 0.35819 0.47555 427.86587 0.753 0.45173

AgeAdolesc:SI110 -0.15765 0.50333 427.86635 -0.313 0.75426

AgeAdlt:SI110 -0.08250 0.50931 427.91732 -0.162 0.87140

AgepAdolesc:SI120 -0.05753 0.47734 427.44307 -0.121 0.90412

AgeAdolesc:SI120 -0.40867 0.50587 428.17449 -0.808 0.41962

AgeAdlt:SI120 -0.61843 0.50932 427.91624 -1.214 0.22533

AgepAdolesc:SI130 0.01112 0.48043 427.58679 0.023 0.98154

AgeAdolesc:SI130 -0.26200 0.50343 427.88010 -0.520 0.60303

AgeAdlt:SI130 -0.48130 0.50932 427.91435 -0.945 0.34521

AgepAdolesc:SI140 0.40251 0.48081 427.68800 0.837 0.40297

AgeAdolesc:SI140 -0.06027 0.50631 427.64231 -0.119 0.90530

AgeAdlt:SI140 -0.51077 0.51438 427.78969 -0.993 0.32129

AgepAdolesc:SI150 0.02828 0.49089 428.01489 0.058 0.95409

AgeAdolesc:SI150 -0.50129 0.51345 427.79938 -0.976 0.32946

AgeAdlt:SI150 -0.63942 0.52185 427.94477 -1.225 0.22113

SI100:HemisR -0.03931 0.35359 428.20699 -0.111 0.91152

SI110:HemisR -0.33306 0.34973 428.55387 -0.952 0.34146

SI120:HemisR -0.11766 0.35084 428.13785 -0.335 0.73751

SI130:HemisR -0.13097 0.35087 427.99247 -0.373 0.70913

SI140:HemisR 0.07197 0.34962 428.26679 0.206 0.83701

SI150:HemisR 0.06764 0.35224 428.31714 0.192 0.84780

---

Signif. codes: 0 ‘***’ 0.001 ‘**’ 0.01 ‘*’ 0.05 ‘.’ 0.1 ‘ ’ 1

===========================================================================

Investigating feature: T1T of ADM

===========================================================================

Linear mixed model fit by maximum likelihood . t-tests use Satterthwaite's method ['lmerModLmerTest']

Formula: curFormula

Data: TMSdev

AIC BIC logLik deviance df.resid

1272.3 1438.0 -596.2 1192.3 425

Scaled residuals:

Min 1Q Median 3Q Max

-3.5371 -0.5982 0.0018 0.6310 3.7972

Random effects:

Groups Name Variance Std.Dev.

ID (Intercept) 1.0438 1.0217

Residual 0.5906 0.7685

Number of obs: 465, groups: ID, 38

Fixed effects:

Estimate Std. Error df t value Pr(>|t|)

(Intercept) 26.47983 0.46886 72.25043 56.478 <2e-16 ***

AgepAdolesc -0.75809 0.60506 69.33819 -1.253 0.2144

AgeAdolesc 1.57908 0.61514 79.50276 2.567 0.0121 *

AgeAdlt 1.12302 0.63052 81.16311 1.781 0.0786 .

SI100 0.13494 0.36370 426.82535 0.371 0.7108

SI110 0.23812 0.34564 426.41563 0.689 0.4913

SI120 -0.38241 0.34582 426.37261 -1.106 0.2694

SI130 0.03459 0.34583 426.35795 0.100 0.9204

SI140 -0.33599 0.34902 426.37627 -0.963 0.3363

SI150 -0.31494 0.35422 426.49008 -0.889 0.3744

HemisR -0.46573 0.28678 429.10634 -1.624 0.1051

AgepAdolesc:HemisR -0.08459 0.22422 429.46680 -0.377 0.7062

AgeAdolesc:HemisR 0.16658 0.22112 429.36859 0.753 0.4517

AgeAdlt:HemisR -0.16274 0.22866 430.23532 -0.712 0.4770

AgepAdolesc:SI100 -0.24029 0.42914 426.91956 -0.560 0.5758

AgeAdolesc:SI100 -0.51760 0.45501 426.92992 -1.138 0.2559

AgeAdlt:SI100 -0.07230 0.46078 426.69560 -0.157 0.8754

AgepAdolesc:SI110 0.17771 0.41875 426.71012 0.424 0.6715

AgeAdolesc:SI110 -0.51822 0.44322 426.68288 -1.169 0.2430

AgeAdlt:SI110 0.18736 0.44849 426.71084 0.418 0.6763

AgepAdolesc:SI120 0.69368 0.42029 426.43637 1.650 0.0996 .

AgeAdolesc:SI120 0.15934 0.44549 426.86711 0.358 0.7208

AgeAdlt:SI120 0.74676 0.44850 426.71024 1.665 0.0966 .

AgepAdolesc:SI130 0.37136 0.42302 426.51947 0.878 0.3805

AgeAdolesc:SI130 0.05627 0.44330 426.69113 0.127 0.8991

AgeAdlt:SI130 0.28528 0.44850 426.70911 0.636 0.5251

AgepAdolesc:SI140 0.71260 0.42337 426.60243 1.683 0.0931 .

AgeAdolesc:SI140 0.36723 0.44581 426.55090 0.824 0.4106

AgeAdlt:SI140 0.53039 0.45294 426.63722 1.171 0.2423

AgepAdolesc:SI150 0.74109 0.43228 426.79292 1.714 0.0872 .

AgeAdolesc:SI150 0.06340 0.45212 426.64124 0.140 0.8885

AgeAdlt:SI150 0.40349 0.45953 426.72650 0.878 0.3804

SI100:HemisR 0.45275 0.31139 426.89737 1.454 0.1467

SI110:HemisR 0.04498 0.30802 427.09567 0.146 0.8840

SI120:HemisR 0.19113 0.30896 426.83969 0.619 0.5365

SI130:HemisR 0.13263 0.30898 426.75268 0.429 0.6680

SI140:HemisR 0.32703 0.30790 426.92871 1.062 0.2888

SI150:HemisR 0.39714 0.31021 426.96057 1.280 0.2012

---

Signif. codes: 0 ‘***’ 0.001 ‘**’ 0.01 ‘*’ 0.05 ‘.’ 0.1 ‘ ’ 1

===========================================================================

Investigating feature: NT of ADM

===========================================================================

Linear mixed model fit by maximum likelihood . t-tests use Satterthwaite's method ['lmerModLmerTest']

Formula: curFormula

Data: TMSdev

AIC BIC logLik deviance df.resid

974.2 1139.8 -447.1 894.2 425

Scaled residuals:

Min 1Q Median 3Q Max

-2.5429 -0.6460 -0.0320 0.5596 3.6054

Random effects:

Groups Name Variance Std.Dev.

ID (Intercept) 0.2072 0.4552

Residual 0.3367 0.5803

Number of obs: 465, groups: ID, 38

Fixed effects:

Estimate Std. Error df t value Pr(>|t|)

(Intercept) 2.502e+00 2.643e-01 1.466e+02 9.465 < 2e-16 ***

AgepAdolesc 1.281e-03 3.380e-01 1.361e+02 0.004 0.99698

AgeAdolesc 5.704e-02 3.535e-01 1.686e+02 0.161 0.87198

AgeAdlt 2.603e-04 3.637e-01 1.731e+02 0.001 0.99943

SI100 1.538e-01 2.744e-01 4.285e+02 0.560 0.57549

SI110 6.450e-01 2.609e-01 4.276e+02 2.472 0.01382 *

SI120 1.153e+00 2.610e-01 4.275e+02 4.416 1.28e-05 ***

SI130 1.338e+00 2.611e-01 4.275e+02 5.126 4.50e-07 ***

SI140 1.411e+00 2.635e-01 4.275e+02 5.358 1.38e-07 ***

SI150 1.315e+00 2.674e-01 4.278e+02 4.918 1.25e-06 ***

HemisR 2.757e-01 2.159e-01 4.339e+02 1.277 0.20236

AgepAdolesc:HemisR -6.993e-01 1.688e-01 4.344e+02 -4.143 4.11e-05 ***

AgeAdolesc:HemisR -4.648e-01 1.665e-01 4.343e+02 -2.792 0.00546 **

AgeAdlt:HemisR -1.751e-01 1.720e-01 4.362e+02 -1.018 0.30917

AgepAdolesc:SI100 2.750e-01 3.238e-01 4.287e+02 0.849 0.39619

AgeAdolesc:SI100 2.111e-02 3.433e-01 4.289e+02 0.061 0.95100

AgeAdlt:SI100 -7.777e-02 3.477e-01 4.283e+02 -0.224 0.82312

AgepAdolesc:SI110 -5.136e-02 3.160e-01 4.283e+02 -0.163 0.87098

AgeAdolesc:SI110 -1.921e-01 3.345e-01 4.283e+02 -0.574 0.56602

AgeAdlt:SI110 -3.871e-01 3.384e-01 4.284e+02 -1.144 0.25339

AgepAdolesc:SI120 -2.780e-01 3.172e-01 4.277e+02 -0.876 0.38134

AgeAdolesc:SI120 -7.563e-01 3.361e-01 4.288e+02 -2.250 0.02496 *

AgeAdlt:SI120 -8.309e-01 3.384e-01 4.284e+02 -2.455 0.01448 *

AgepAdolesc:SI130 -1.333e-01 3.193e-01 4.279e+02 -0.418 0.67651

AgeAdolesc:SI130 -5.981e-01 3.345e-01 4.283e+02 -1.788 0.07448 .

AgeAdlt:SI130 -9.031e-01 3.384e-01 4.284e+02 -2.668 0.00791 **

AgepAdolesc:SI140 -1.264e-01 3.195e-01 4.280e+02 -0.396 0.69262

AgeAdolesc:SI140 -5.233e-01 3.365e-01 4.280e+02 -1.555 0.12060

AgeAdlt:SI140 -9.200e-01 3.418e-01 4.282e+02 -2.692 0.00739 **

AgepAdolesc:SI150 9.561e-02 3.262e-01 4.285e+02 0.293 0.76959

AgeAdolesc:SI150 -2.236e-01 3.412e-01 4.282e+02 -0.655 0.51258

AgeAdlt:SI150 -8.095e-01 3.468e-01 4.284e+02 -2.334 0.02003 *

SI100:HemisR 3.282e-01 2.349e-01 4.288e+02 1.397 0.16319

SI110:HemisR 1.929e-01 2.324e-01 4.293e+02 0.830 0.40689

SI120:HemisR 6.942e-02 2.331e-01 4.287e+02 0.298 0.76601

SI130:HemisR 2.455e-02 2.331e-01 4.285e+02 0.105 0.91620

SI140:HemisR -1.682e-02 2.323e-01 4.289e+02 -0.072 0.94232

SI150:HemisR 7.980e-02 2.340e-01 4.290e+02 0.341 0.73330

---

Signif. codes: 0 ‘***’ 0.001 ‘**’ 0.01 ‘*’ 0.05 ‘.’ 0.1 ‘ ’ 1

===========================================================================

Investigating feature: Amp of FDI

===========================================================================

Linear mixed model fit by maximum likelihood . t-tests use Satterthwaite's method ['lmerModLmerTest']

Formula: curFormula

Data: TMSdev

AIC BIC logLik deviance df.resid

86.9 253.6 -3.4 6.9 438

Scaled residuals:

Min 1Q Median 3Q Max

-3.4788 -0.5350 0.0132 0.6369 3.0275

Random effects:

Groups Name Variance Std.Dev.

ID (Intercept) 0.02173 0.1474

Residual 0.05134 0.2266

Number of obs: 478, groups: ID, 38

Fixed effects:

Estimate Std. Error df t value Pr(>|t|)

(Intercept) 1.95640 0.09830 208.90136 19.903 < 2e-16 ***

AgepAdolesc 0.20879 0.12743 205.76940 1.638 0.102866

AgeAdolesc 0.08022 0.12217 193.42922 0.657 0.512229

AgeAdlt 0.31982 0.12135 178.09897 2.636 0.009141 **

SI100 0.24195 0.10545 443.41923 2.295 0.022227 *

SI110 0.37506 0.10301 442.49350 3.641 0.000304 ***

SI120 0.49185 0.10299 442.57702 4.776 2.44e-06 ***

SI130 0.62022 0.10301 442.49497 6.021 3.64e-09 ***

SI140 0.69371 0.10297 442.51097 6.737 5.05e-11 ***

SI150 0.80757 0.10293 442.49809 7.846 3.25e-14 ***

HemisR 0.04451 0.07814 445.91951 0.570 0.569224

AgepAdolesc:HemisR 0.11099 0.06405 448.73871 1.733 0.083786 .

AgeAdolesc:HemisR 0.07441 0.06295 449.09000 1.182 0.237798

AgeAdlt:HemisR 0.03910 0.06539 454.21503 0.598 0.550167

AgepAdolesc:SI100 0.04050 0.12843 442.93286 0.315 0.752621

AgeAdolesc:SI100 0.19391 0.12556 442.24445 1.544 0.123237

AgeAdlt:SI100 0.02042 0.12422 442.72680 0.164 0.869473

AgepAdolesc:SI110 0.14993 0.12707 442.51106 1.180 0.238655

AgeAdolesc:SI110 0.41445 0.12359 441.74475 3.353 0.000867 ***

AgeAdlt:SI110 0.25533 0.12283 442.26640 2.079 0.038223 *

AgepAdolesc:SI120 0.21038 0.12707 442.51177 1.656 0.098501 .

AgeAdolesc:SI120 0.46000 0.12298 441.32091 3.740 0.000208 ***

AgeAdlt:SI120 0.40533 0.12283 442.26674 3.300 0.001046 **

AgepAdolesc:SI130 0.26782 0.12799 441.85215 2.092 0.036967 *

AgeAdolesc:SI130 0.60081 0.12251 441.26197 4.904 1.32e-06 ***

AgeAdlt:SI130 0.46648 0.12283 442.26641 3.798 0.000166 ***

AgepAdolesc:SI140 0.26589 0.12749 442.03880 2.086 0.037592 *

AgeAdolesc:SI140 0.58828 0.12251 441.26204 4.802 2.16e-06 ***

AgeAdlt:SI140 0.50232 0.12283 442.26674 4.089 5.14e-05 ***

AgepAdolesc:SI150 0.16783 0.12707 442.51117 1.321 0.187238

AgeAdolesc:SI150 0.53781 0.12251 441.26201 4.390 1.42e-05 ***

AgeAdlt:SI150 0.42509 0.12283 442.26649 3.461 0.000591 ***

SI100:HemisR -0.13676 0.08555 442.94744 -1.599 0.110631

SI110:HemisR -0.06521 0.08505 442.95783 -0.767 0.443628

SI120:HemisR -0.06409 0.08492 443.46008 -0.755 0.450800

SI130:HemisR -0.09765 0.08504 442.96783 -1.148 0.251451

SI140:HemisR -0.11896 0.08484 443.07743 -1.402 0.161568

SI150:HemisR -0.10270 0.08459 442.99881 -1.214 0.225373

---

Signif. codes: 0 ‘***’ 0.001 ‘**’ 0.01 ‘*’ 0.05 ‘.’ 0.1 ‘ ’ 1

===========================================================================

Investigating feature: iDur of FDI

===========================================================================

Linear mixed model fit by maximum likelihood . t-tests use Satterthwaite's method ['lmerModLmerTest']

Formula: curFormula

Data: TMSdev

AIC BIC logLik deviance df.resid

3043.1 3209.9 -1481.6 2963.1 438

Scaled residuals:

Min 1Q Median 3Q Max

-2.9493 -0.5554 0.0187 0.6345 3.3886

Random effects:

Groups Name Variance Std.Dev.

ID (Intercept) 8.486 2.913

Residual 25.295 5.029

Number of obs: 478, groups: ID, 38

Fixed effects:

Estimate Std. Error df t value Pr(>|t|)

(Intercept) 18.9704 2.1072 241.8060 9.003 < 2e-16 ***

AgepAdolesc 5.9252 2.7307 238.3117 2.170 0.031009 *

AgeAdolesc 0.6845 2.6139 224.7947 0.262 0.793651

AgeAdlt 4.1010 2.5907 206.6699 1.583 0.114953

SI100 0.3075 2.3396 443.1908 0.131 0.895489

SI110 2.5821 2.2857 442.0809 1.130 0.259240

SI120 4.8942 2.2852 442.1803 2.142 0.032764 *

SI130 8.2428 2.2857 442.0827 3.606 0.000346 ***

SI140 9.2195 2.2849 442.1019 4.035 6.43e-05 ***

SI150 12.3879 2.2839 442.0864 5.424 9.61e-08 ***

HemisR -1.8548 1.7331 446.1385 -1.070 0.285115

AgepAdolesc:HemisR 2.7381 1.4201 449.2552 1.928 0.054467 .

AgeAdolesc:HemisR 1.2388 1.3958 449.6693 0.888 0.375256

AgeAdlt:HemisR 1.6714 1.4489 455.6356 1.154 0.249268

AgepAdolesc:SI100 0.3190 2.8497 442.6567 0.112 0.910925

AgeAdolesc:SI100 3.2898 2.7863 441.7978 1.181 0.238359

AgeAdlt:SI100 1.3268 2.7563 442.3816 0.481 0.630501

AgepAdolesc:SI110 3.9083 2.8195 442.1513 1.386 0.166404

AgeAdolesc:SI110 8.2591 2.7427 441.1968 3.011 0.002751 **

AgeAdlt:SI110 4.5793 2.7257 441.8291 1.680 0.093658 .

AgepAdolesc:SI120 3.2141 2.8195 442.1521 1.140 0.254928

AgeAdolesc:SI120 9.4693 2.7293 440.6915 3.469 0.000573 ***

AgeAdlt:SI120 6.7482 2.7257 441.8296 2.476 0.013668 *

AgepAdolesc:SI130 4.1253 2.8404 441.3574 1.452 0.147108

AgeAdolesc:SI130 9.4965 2.7189 440.6216 3.493 0.000526 ***

AgeAdlt:SI130 6.3428 2.7257 441.8292 2.327 0.020416 *

AgepAdolesc:SI140 3.4487 2.8291 441.5826 1.219 0.223489

AgeAdolesc:SI140 9.4289 2.7189 440.6217 3.468 0.000576 ***

AgeAdlt:SI140 5.4091 2.7257 441.8296 1.984 0.047819 *

AgepAdolesc:SI150 0.6028 2.8195 442.1514 0.214 0.830817

AgeAdolesc:SI150 7.8081 2.7189 440.6217 2.872 0.004279 **

AgeAdlt:SI150 3.7960 2.7257 441.8293 1.393 0.164412

SI100:HemisR 0.3575 1.8982 442.6932 0.188 0.850695

SI110:HemisR 0.9518 1.8871 442.7109 0.504 0.614242

SI120:HemisR 2.8539 1.8841 443.3085 1.515 0.130551

SI130:HemisR 3.0702 1.8868 442.7232 1.627 0.104404

SI140:HemisR 3.3557 1.8825 442.8553 1.783 0.075334 .

SI150:HemisR 2.0041 1.8770 442.7610 1.068 0.286227

---

Signif. codes: 0 ‘***’ 0.001 ‘**’ 0.01 ‘*’ 0.05 ‘.’ 0.1 ‘ ’ 1

===========================================================================

Investigating feature: Lat of FDI

===========================================================================

Linear mixed model fit by maximum likelihood . t-tests use Satterthwaite's method ['lmerModLmerTest']

Formula: curFormula

Data: TMSdev

AIC BIC logLik deviance df.resid

1215.0 1381.8 -567.5 1135.0 438

Scaled residuals:

Min 1Q Median 3Q Max

-4.1037 -0.5887 -0.0777 0.5346 4.6809

Random effects:

Groups Name Variance Std.Dev.

ID (Intercept) 0.9224 0.9604

Residual 0.4881 0.6986

Number of obs: 478, groups: ID, 38

Fixed effects:

Estimate Std. Error df t value Pr(>|t|)

(Intercept) 22.81083 0.44086 73.83208 51.742 < 2e-16 ***

AgepAdolesc -0.98939 0.57322 73.07280 -1.726 0.088573 .

AgeAdolesc 2.00814 0.55625 70.10152 3.610 0.000571 ***

AgeAdlt 1.38484 0.56051 67.05702 2.471 0.016035 *

SI100 0.22696 0.32562 440.51716 0.697 0.486165

SI110 -0.39780 0.31796 440.24481 -1.251 0.211562

SI120 -0.65632 0.31790 440.26974 -2.065 0.039552 *

SI130 -0.95527 0.31795 440.24519 -3.004 0.002812 **

SI140 -1.32241 0.31784 440.24977 -4.161 3.82e-05 ***

SI150 -1.80750 0.31770 440.24610 -5.689 2.33e-08 ***

HemisR -0.05971 0.24158 441.29856 -0.247 0.804903

AgepAdolesc:HemisR -0.15421 0.19832 442.38871 -0.778 0.437222

AgeAdolesc:HemisR -0.35795 0.19496 442.49982 -1.836 0.067035 .

AgeAdlt:HemisR -0.06256 0.20307 444.17296 -0.308 0.758188

AgepAdolesc:SI100 0.32926 0.39649 440.32641 0.830 0.406746

AgeAdolesc:SI100 -0.43790 0.38753 440.15593 -1.130 0.259108

AgeAdlt:SI100 0.04167 0.38347 440.29139 0.109 0.913522

AgepAdolesc:SI110 0.48463 0.39220 440.20211 1.236 0.217248

AgeAdolesc:SI110 -0.18502 0.38135 440.01075 -0.485 0.627801

AgeAdlt:SI110 0.43155 0.37911 440.15642 1.138 0.255600

AgepAdolesc:SI120 0.59192 0.39220 440.20232 1.509 0.131963

AgeAdolesc:SI120 0.09632 0.37941 439.88528 0.254 0.799720

AgeAdlt:SI120 0.40815 0.37910 440.15652 1.077 0.282243

AgepAdolesc:SI130 0.57627 0.39495 440.01445 1.459 0.145251

AgeAdolesc:SI130 -0.04861 0.37794 439.86747 -0.129 0.897725

AgeAdlt:SI130 0.38058 0.37911 440.15643 1.004 0.315982

AgepAdolesc:SI140 0.88996 0.39343 440.06741 2.262 0.024180 *

AgeAdolesc:SI140 0.34580 0.37794 439.86749 0.915 0.360720

AgeAdlt:SI140 0.79246 0.37910 440.15652 2.090 0.037159 *

AgepAdolesc:SI150 1.13365 0.39220 440.20215 2.890 0.004037 **

AgeAdolesc:SI150 0.58318 0.37794 439.86748 1.543 0.123542

AgeAdlt:SI150 1.12439 0.37910 440.15645 2.966 0.003182 **

SI100:HemisR -0.37740 0.26411 440.31300 -1.429 0.153724

SI110:HemisR -0.24463 0.26256 440.31093 -0.932 0.352014

SI120:HemisR -0.25617 0.26222 440.46082 -0.977 0.329137

SI130:HemisR -0.16343 0.26252 440.31357 -0.623 0.533918

SI140:HemisR -0.26797 0.26194 440.34480 -1.023 0.306847

SI150:HemisR -0.19527 0.26116 440.32202 -0.748 0.455056

---

Signif. codes: 0 ‘***’ 0.001 ‘**’ 0.01 ‘*’ 0.05 ‘.’ 0.1 ‘ ’ 1

===========================================================================

Investigating feature: T1T of FDI

===========================================================================

Linear mixed model fit by maximum likelihood . t-tests use Satterthwaite's method ['lmerModLmerTest']

Formula: curFormula

Data: TMSdev

AIC BIC logLik deviance df.resid

1249.4 1416.1 -584.7 1169.4 438

Scaled residuals:

Min 1Q Median 3Q Max

-2.8252 -0.5220 -0.0222 0.5298 4.1406

Random effects:

Groups Name Variance Std.Dev.

ID (Intercept) 1.1191 1.0579

Residual 0.5192 0.7206

Number of obs: 478, groups: ID, 38

Fixed effects:

Estimate Std. Error df t value Pr(>|t|)

(Intercept) 26.109420 0.475876 69.081995 54.866 < 2e-16 ***

AgepAdolesc -1.339762 0.618902 68.425992 -2.165 0.033899 *

AgeAdolesc 1.589461 0.601153 65.861310 2.644 0.010230 *

AgeAdlt 1.305311 0.606389 63.244159 2.153 0.035173 *

SI100 -0.262045 0.335861 440.431834 -0.780 0.435680

SI110 -0.380318 0.327951 440.190586 -1.160 0.246808

SI120 -0.374674 0.327891 440.212674 -1.143 0.253793

SI130 -0.387252 0.327942 440.190921 -1.181 0.238297

SI140 -0.908921 0.327831 440.194972 -2.773 0.005798 **

SI150 -0.957515 0.327684 440.191729 -2.922 0.003656 **

HemisR -0.553470 0.249191 441.125671 -2.221 0.026853 *

AgepAdolesc:HemisR 0.275122 0.204580 442.101699 1.345 0.179375

AgeAdolesc:HemisR -0.013413 0.201123 442.200430 -0.067 0.946859

AgeAdlt:HemisR 0.272112 0.209506 443.688328 1.299 0.194678

AgepAdolesc:SI100 0.718602 0.408954 440.260986 1.757 0.079584 .

AgeAdolesc:SI100 0.135352 0.399708 440.111225 0.339 0.735052

AgeAdlt:SI100 0.455340 0.395521 440.230930 1.151 0.250258

AgepAdolesc:SI110 0.934305 0.404530 440.150888 2.310 0.021372 *

AgeAdolesc:SI110 0.354757 0.393332 439.982704 0.902 0.367589

AgeAdlt:SI110 0.616313 0.391018 440.111398 1.576 0.115704

AgepAdolesc:SI120 1.085942 0.404529 440.151074 2.684 0.007539 **

AgeAdolesc:SI120 0.599266 0.391322 439.871589 1.531 0.126392

AgeAdlt:SI120 0.624870 0.391015 440.111484 1.598 0.110745

AgepAdolesc:SI130 1.318166 0.407358 439.984976 3.236 0.001304 **

AgeAdolesc:SI130 0.639559 0.389811 439.855803 1.641 0.101577

AgeAdlt:SI130 0.688604 0.391018 440.111402 1.761 0.078924 .

AgepAdolesc:SI140 1.597726 0.405789 440.031789 3.937 9.58e-05 ***

AgeAdolesc:SI140 1.036313 0.389811 439.855821 2.659 0.008135 **

AgeAdlt:SI140 1.099561 0.391015 440.111486 2.812 0.005143 **

AgepAdolesc:SI150 1.408539 0.404528 440.150919 3.482 0.000548 ***

AgeAdolesc:SI150 1.210900 0.389810 439.855817 3.106 0.002016 **

AgeAdlt:SI150 1.154662 0.391012 440.111424 2.953 0.003315 **

SI100:HemisR 0.219920 0.272412 440.248438 0.807 0.419926

SI110:HemisR 0.177059 0.270817 440.246407 0.654 0.513585

SI120:HemisR 0.041498 0.270462 440.379181 0.153 0.878126

SI130:HemisR -0.064583 0.270773 440.248731 -0.239 0.811594

SI140:HemisR 0.100700 0.270171 440.276342 0.373 0.709532

SI150:HemisR -0.004013 0.269373 440.256184 -0.015 0.988120

---

Signif. codes: 0 ‘***’ 0.001 ‘**’ 0.01 ‘*’ 0.05 ‘.’ 0.1 ‘ ’ 1

===========================================================================

Investigating feature: NT of FDI

===========================================================================

Linear mixed model fit by maximum likelihood . t-tests use Satterthwaite's method ['lmerModLmerTest']

Formula: curFormula

Data: TMSdev

AIC BIC logLik deviance df.resid

814.6 981.3 -367.3 734.6 438

Scaled residuals:

Min 1Q Median 3Q Max

-2.7502 -0.6129 -0.0964 0.5100 3.7052

Random effects:

Groups Name Variance Std.Dev.

ID (Intercept) 0.1426 0.3777

Residual 0.2292 0.4787

Number of obs: 478, groups: ID, 38

Fixed effects:

Estimate Std. Error df t value Pr(>|t|)

(Intercept) 2.22761 0.22291 157.37852 9.993 < 2e-16 ***

AgepAdolesc 0.09461 0.28920 154.98859 0.327 0.743994

AgeAdolesc -0.22006 0.27807 145.51317 -0.791 0.430005

AgeAdlt -0.22281 0.27726 134.76133 -0.804 0.423036

SI100 0.78891 0.22291 442.66301 3.539 0.000444 ***

SI110 1.11221 0.21772 441.97026 5.108 4.84e-07 ***

SI120 1.26808 0.21768 442.03324 5.826 1.10e-08 ***

SI130 1.49899 0.21771 441.97131 6.885 1.99e-11 ***

SI140 1.80072 0.21764 441.98319 8.274 1.54e-15 ***

SI150 1.87937 0.21754 441.97364 8.639 < 2e-16 ***

HemisR 0.40136 0.16525 444.57752 2.429 0.015545 *

AgepAdolesc:HemisR -0.03907 0.13552 446.92431 -0.288 0.773259

AgeAdolesc:HemisR -0.12223 0.13321 447.19403 -0.918 0.359367

AgeAdlt:HemisR 0.13792 0.13852 451.18772 0.996 0.319919

AgepAdolesc:SI100 -0.68414 0.27147 442.25536 -2.520 0.012082 *

AgeAdolesc:SI100 -0.33363 0.26538 441.76984 -1.257 0.209359

AgeAdlt:SI100 -0.49009 0.26256 442.12534 -1.867 0.062627 .

AgepAdolesc:SI110 -0.81658 0.26857 441.93947 -3.040 0.002502 **

AgeAdolesc:SI110 -0.64208 0.26119 441.39757 -2.458 0.014342 *

AgeAdlt:SI110 -0.68465 0.25961 441.78126 -2.637 0.008654 **

AgepAdolesc:SI120 -0.82625 0.26857 441.94001 -3.077 0.002224 **

AgeAdolesc:SI120 -0.59230 0.25989 441.07884 -2.279 0.023141 *

AgeAdlt:SI120 -0.79308 0.25961 441.78151 -3.055 0.002388 **

AgepAdolesc:SI130 -0.91985 0.27050 441.45100 -3.401 0.000734 ***

AgeAdolesc:SI130 -0.72323 0.25889 441.03416 -2.794 0.005440 **

AgeAdlt:SI130 -0.96697 0.25961 441.78127 -3.725 0.000221 ***

AgepAdolesc:SI140 -1.04298 0.26944 441.58916 -3.871 0.000125 ***

AgeAdolesc:SI140 -0.94998 0.25889 441.03421 -3.669 0.000273 ***

AgeAdlt:SI140 -1.20847 0.25961 441.78151 -4.655 4.29e-06 ***

AgepAdolesc:SI150 -0.96315 0.26857 441.93956 -3.586 0.000373 ***

AgeAdolesc:SI150 -0.77085 0.25889 441.03419 -2.978 0.003066 **

AgeAdlt:SI150 -1.24727 0.25961 441.78133 -4.804 2.13e-06 ***

SI100:HemisR -0.36126 0.18083 442.24968 -1.998 0.046356 *

SI110:HemisR -0.45999 0.17977 442.25277 -2.559 0.010838 *

SI120:HemisR -0.52939 0.17951 442.63163 -2.949 0.003356 **

SI130:HemisR -0.45302 0.17974 442.25998 -2.520 0.012075 *

SI140:HemisR -0.35843 0.17934 442.34124 -1.999 0.046261 *

SI150:HemisR -0.27218 0.17881 442.28255 -1.522 0.128685

---

Signif. codes: 0 ‘***’ 0.001 ‘**’ 0.01 ‘*’ 0.05 ‘.’ 0.1 ‘ ’ 1

===========================================================================

Investigating feature: Amp of ECR

===========================================================================

Linear mixed model fit by maximum likelihood . t-tests use Satterthwaite's method ['lmerModLmerTest']

Formula: curFormula

Data: TMSdev

AIC BIC logLik deviance df.resid

-8.2 158.4 44.1 -88.2 436

Scaled residuals:

Min 1Q Median 3Q Max

-3.6946 -0.6344 0.0325 0.6417 3.9533

Random effects:

Groups Name Variance Std.Dev.

ID (Intercept) 0.02553 0.1598

Residual 0.04092 0.2023

Number of obs: 476, groups: ID, 38

Fixed effects:

Estimate Std. Error df t value Pr(>|t|)

(Intercept) 2.095619 0.094649 157.949171 22.141 < 2e-16 ***

AgepAdolesc 0.087959 0.119060 140.214333 0.739 0.461276

AgeAdolesc 0.028192 0.121855 160.938057 0.231 0.817332

AgeAdlt -0.069539 0.123714 157.595327 -0.562 0.574849

SI100 0.220588 0.094660 438.314344 2.330 0.020242 *

SI110 0.333421 0.092434 438.754002 3.607 0.000345 ***

SI120 0.409648 0.092396 438.748772 4.434 1.17e-05 ***

SI130 0.518181 0.092396 438.748772 5.608 3.62e-08 ***

SI140 0.530614 0.092396 438.748772 5.743 1.74e-08 ***

SI150 0.607764 0.092396 438.748772 6.578 1.36e-10 ***

HemisR 0.096823 0.072591 444.689841 1.334 0.182950

AgepAdolesc:HemisR 0.005413 0.057355 445.307232 0.094 0.924847

AgeAdolesc:HemisR -0.153731 0.056747 445.580803 -2.709 0.007007 **

AgeAdlt:HemisR -0.055834 0.058532 447.688774 -0.954 0.340643

AgepAdolesc:SI100 0.004629 0.113607 438.078549 0.041 0.967518

AgeAdolesc:SI100 -0.020076 0.116680 439.349693 -0.172 0.863466

AgeAdlt:SI100 0.122318 0.118357 439.396391 1.033 0.301953

AgepAdolesc:SI110 -0.019920 0.110608 439.137050 -0.180 0.857162

AgeAdolesc:SI110 0.048287 0.114711 439.673752 0.421 0.674001

AgeAdlt:SI110 0.109425 0.115784 440.036183 0.945 0.345137

AgepAdolesc:SI120 0.071710 0.110608 439.137050 0.648 0.517114

AgeAdolesc:SI120 0.185572 0.114156 439.321379 1.626 0.104751

AgeAdlt:SI120 0.223451 0.115784 440.036230 1.930 0.054262 .

AgepAdolesc:SI130 0.126533 0.110608 439.137050 1.144 0.253256

AgeAdolesc:SI130 0.238869 0.114156 439.321379 2.092 0.036969 *

AgeAdlt:SI130 0.264907 0.115784 440.036230 2.288 0.022614 *

AgepAdolesc:SI140 0.139997 0.110608 439.137050 1.266 0.206289

AgeAdolesc:SI140 0.295654 0.114156 439.321379 2.590 0.009919 **

AgeAdlt:SI140 0.354147 0.115784 440.036230 3.059 0.002359 **

AgepAdolesc:SI150 0.122636 0.110608 439.137050 1.109 0.268145

AgeAdolesc:SI150 0.282504 0.114156 439.321379 2.475 0.013710 *

AgeAdlt:SI150 0.332044 0.115784 440.036230 2.868 0.004332 **

SI100:HemisR -0.137964 0.080471 439.173929 -1.714 0.087151 .

SI110:HemisR 0.059065 0.077986 439.205143 0.757 0.449228

SI120:HemisR 0.042583 0.077784 439.178311 0.547 0.584343

SI130:HemisR -0.011668 0.077784 439.178311 -0.150 0.880829

SI140:HemisR -0.028273 0.077784 439.178311 -0.363 0.716422

SI150:HemisR -0.036696 0.077784 439.178311 -0.472 0.637325

---

Signif. codes: 0 ‘***’ 0.001 ‘**’ 0.01 ‘*’ 0.05 ‘.’ 0.1 ‘ ’ 1

===========================================================================

Investigating feature: iDur of ECR

===========================================================================

Linear mixed model fit by maximum likelihood . t-tests use Satterthwaite's method ['lmerModLmerTest']

Formula: curFormula

Data: TMSdev

AIC BIC logLik deviance df.resid

2834.5 3001.1 -1377.3 2754.5 436

Scaled residuals:

Min 1Q Median 3Q Max

-2.91048 -0.67485 -0.05557 0.56965 3.06481

Random effects:

Groups Name Variance Std.Dev.

ID (Intercept) 14.60 3.822

Residual 15.59 3.949

Number of obs: 476, groups: ID, 38

Fixed effects:

Estimate Std. Error df t value Pr(>|t|)

(Intercept) 20.295274 2.027687 116.608609 10.009 < 2e-16 ***

AgepAdolesc 1.468081 2.566800 104.885299 0.572 0.56858

AgeAdolesc 0.009166 2.607890 118.730988 0.004 0.99720

AgeAdlt 0.866758 2.650252 116.956936 0.327 0.74422

SI100 2.218901 1.847927 438.096169 1.201 0.23050

SI110 3.349921 1.804621 438.406750 1.856 0.06408 .

SI120 6.059635 1.803873 438.403109 3.359 0.00085 ***

SI130 8.174262 1.803873 438.403109 4.532 7.56e-06 ***

SI140 9.505446 1.803873 438.403109 5.269 2.15e-07 ***

SI150 11.582467 1.803873 438.403108 6.421 3.53e-10 ***

HemisR 2.007257 1.418782 442.760238 1.415 0.15784

AgepAdolesc:HemisR 0.407641 1.121132 443.259198 0.364 0.71633

AgeAdolesc:HemisR -0.668619 1.109295 443.456678 -0.603 0.54699

AgeAdlt:HemisR -2.079287 1.144679 445.077212 -1.816 0.06997 .

AgepAdolesc:SI100 0.780288 2.217716 437.930680 0.352 0.72513

AgeAdolesc:SI100 0.317469 2.278216 438.817314 0.139 0.88924

AgeAdlt:SI100 2.348506 2.310976 438.860926 1.016 0.31008

AgepAdolesc:SI110 2.490080 2.159575 438.674386 1.153 0.24952

AgeAdolesc:SI110 1.631688 2.239904 439.046137 0.728 0.46672

AgeAdlt:SI110 4.232333 2.261003 439.309413 1.872 0.06189 .

AgepAdolesc:SI120 3.236056 2.159573 438.674386 1.498 0.13473

AgeAdolesc:SI120 3.559784 2.228913 438.799516 1.597 0.11096

AgeAdlt:SI120 5.238083 2.260997 439.309447 2.317 0.02098 *

AgepAdolesc:SI130 3.796442 2.159573 438.674386 1.758 0.07945 .

AgeAdolesc:SI130 4.272669 2.228913 438.799516 1.917 0.05590 .

AgeAdlt:SI130 5.613995 2.260997 439.309447 2.483 0.01340 *

AgepAdolesc:SI140 4.035041 2.159573 438.674386 1.868 0.06237 .

AgeAdolesc:SI140 5.938677 2.228913 438.799516 2.664 0.00800 **

AgeAdlt:SI140 5.928898 2.260997 439.309447 2.622 0.00904 **

AgepAdolesc:SI150 3.234626 2.159573 438.674386 1.498 0.13490

AgeAdolesc:SI150 6.737217 2.228913 438.799516 3.023 0.00265 **

AgeAdlt:SI150 5.746726 2.260997 439.309447 2.542 0.01137 *

SI100:HemisR -1.078709 1.571180 438.697247 -0.687 0.49272

SI110:HemisR 0.759390 1.522660 438.718930 0.499 0.61822

SI120:HemisR 0.140008 1.518703 438.700223 0.092 0.92659

SI130:HemisR 1.024983 1.518703 438.700223 0.675 0.50009

SI140:HemisR -1.197581 1.518703 438.700223 -0.789 0.43080

SI150:HemisR -1.517793 1.518703 438.700223 -0.999 0.31815

---

Signif. codes: 0 ‘***’ 0.001 ‘**’ 0.01 ‘*’ 0.05 ‘.’ 0.1 ‘ ’ 1

===========================================================================

Investigating feature: Lat of ECR

===========================================================================

Linear mixed model fit by maximum likelihood . t-tests use Satterthwaite's method ['lmerModLmerTest']

Formula: curFormula

Data: TMSdev

AIC BIC logLik deviance df.resid

1425.8 1592.5 -672.9 1345.8 436

Scaled residuals:

Min 1Q Median 3Q Max

-2.9266 -0.5779 -0.0156 0.5453 3.6919

Random effects:

Groups Name Variance Std.Dev.

ID (Intercept) 0.4886 0.6990

Residual 0.8363 0.9145

Number of obs: 476, groups: ID, 38

Fixed effects:

Estimate Std. Error df t value Pr(>|t|)

(Intercept) 19.05120 0.42229 167.70569 45.114 < 2e-16 ***

AgepAdolesc -1.81552 0.53068 148.77170 -3.421 0.000805 ***

AgeAdolesc 0.20262 0.54376 170.84301 0.373 0.709886

AgeAdlt -0.09262 0.55196 167.13862 -0.168 0.866934

SI100 -0.38817 0.42791 438.89624 -0.907 0.364839

SI110 -0.97181 0.41784 439.35423 -2.326 0.020485 *

SI120 -1.66627 0.41767 439.34877 -3.989 7.76e-05 ***

SI130 -2.03185 0.41767 439.34877 -4.865 1.60e-06 ***

SI140 -2.23056 0.41767 439.34877 -5.340 1.49e-07 ***

SI150 -2.51470 0.41767 439.34877 -6.021 3.67e-09 ***

HemisR -0.81498 0.32808 445.49195 -2.484 0.013354 *

AgepAdolesc:HemisR 0.37352 0.25921 446.11762 1.441 0.150290

AgeAdolesc:HemisR 0.36892 0.25646 446.40130 1.439 0.150992

AgeAdlt:HemisR 0.81597 0.26451 448.56071 3.085 0.002162 **

AgepAdolesc:SI100 0.72566 0.51357 438.65030 1.413 0.158368

AgeAdolesc:SI100 0.44950 0.52743 439.97751 0.852 0.394541

AgeAdlt:SI100 0.52446 0.53501 440.02329 0.980 0.327492

AgepAdolesc:SI110 0.87942 0.49999 439.75398 1.759 0.079295 .

AgeAdolesc:SI110 0.98733 0.51853 440.31506 1.904 0.057548 .

AgeAdlt:SI110 0.79950 0.52337 440.69060 1.528 0.127332

AgepAdolesc:SI120 1.20455 0.49999 439.75398 2.409 0.016400 *

AgeAdolesc:SI120 0.98938 0.51602 439.94741 1.917 0.055844 .

AgeAdlt:SI120 1.09519 0.52337 440.69065 2.093 0.036959 *

AgepAdolesc:SI130 0.80358 0.49999 439.75398 1.607 0.108729

AgeAdolesc:SI130 0.76665 0.51602 439.94741 1.486 0.138079

AgeAdlt:SI130 0.89809 0.52337 440.69065 1.716 0.086870 .

AgepAdolesc:SI140 1.03452 0.49999 439.75398 2.069 0.039121 *

AgeAdolesc:SI140 0.81418 0.51602 439.94741 1.578 0.115331

AgeAdlt:SI140 0.93810 0.52337 440.69065 1.792 0.073753 .

AgepAdolesc:SI150 1.03284 0.49999 439.75398 2.066 0.039439 *

AgeAdolesc:SI150 0.74868 0.51602 439.94741 1.451 0.147529

AgeAdlt:SI150 1.10927 0.52337 440.69065 2.119 0.034610 *

SI100:HemisR -0.04480 0.36376 439.79336 -0.123 0.902043

SI110:HemisR -0.24111 0.35252 439.82598 -0.684 0.494358

SI120:HemisR -0.00145 0.35161 439.79796 -0.004 0.996712

SI130:HemisR 0.18063 0.35161 439.79796 0.514 0.607697

SI140:HemisR 0.24117 0.35161 439.79796 0.686 0.493129

SI150:HemisR 0.26682 0.35161 439.79796 0.759 0.448351

---

Signif. codes: 0 ‘***’ 0.001 ‘**’ 0.01 ‘*’ 0.05 ‘.’ 0.1 ‘ ’ 1

===========================================================================

Investigating feature: T1T of ECR

===========================================================================

Linear mixed model fit by maximum likelihood . t-tests use Satterthwaite's method ['lmerModLmerTest']

Formula: curFormula

Data: TMSdev

AIC BIC logLik deviance df.resid

1703.1 1869.7 -811.5 1623.1 436

Scaled residuals:

Min 1Q Median 3Q Max

-3.1128 -0.6683 -0.1025 0.6313 3.2788

Random effects:

Groups Name Variance Std.Dev.

ID (Intercept) 1.366 1.169

Residual 1.446 1.203

Number of obs: 476, groups: ID, 38

Fixed effects:

Estimate Std. Error df t value Pr(>|t|)

(Intercept) 2.343e+01 6.189e-01 1.164e+02 37.856 < 2e-16 ***

AgepAdolesc -8.548e-01 7.836e-01 1.048e+02 -1.091 0.27779

AgeAdolesc -5.493e-01 7.960e-01 1.185e+02 -0.690 0.49149

AgeAdlt 3.666e-01 8.089e-01 1.168e+02 0.453 0.65125

SI100 1.046e-04 5.629e-01 4.383e+02 0.000 0.99985

SI110 -1.265e-01 5.497e-01 4.386e+02 -0.230 0.81805

SI120 -6.648e-01 5.494e-01 4.386e+02 -1.210 0.22697

SI130 -5.944e-01 5.494e-01 4.386e+02 -1.082 0.27992

SI140 -6.961e-01 5.494e-01 4.386e+02 -1.267 0.20584

SI150 -9.422e-01 5.494e-01 4.386e+02 -1.715 0.08710 .

HemisR -6.453e-01 4.322e-01 4.429e+02 -1.493 0.13611

AgepAdolesc:HemisR 4.414e-01 3.415e-01 4.434e+02 1.293 0.19683

AgeAdolesc:HemisR 9.725e-01 3.379e-01 4.436e+02 2.878 0.00419 **

AgeAdlt:HemisR 2.637e-01 3.487e-01 4.452e+02 0.756 0.44985

AgepAdolesc:SI100 -4.116e-02 6.755e-01 4.381e+02 -0.061 0.95144

AgeAdolesc:SI100 4.990e-01 6.939e-01 4.390e+02 0.719 0.47246

AgeAdlt:SI100 -5.367e-02 7.039e-01 4.390e+02 -0.076 0.93926

AgepAdolesc:SI110 1.344e-01 6.578e-01 4.389e+02 0.204 0.83820

AgeAdolesc:SI110 5.556e-01 6.823e-01 4.392e+02 0.814 0.41586

AgeAdlt:SI110 1.499e-02 6.887e-01 4.395e+02 0.022 0.98264

AgepAdolesc:SI120 2.655e-01 6.578e-01 4.389e+02 0.404 0.68663

AgeAdolesc:SI120 7.623e-01 6.789e-01 4.390e+02 1.123 0.26215

AgeAdlt:SI120 -2.160e-02 6.887e-01 4.395e+02 -0.031 0.97499

AgepAdolesc:SI130 -1.378e-01 6.578e-01 4.389e+02 -0.209 0.83422

AgeAdolesc:SI130 4.946e-01 6.789e-01 4.390e+02 0.729 0.46664

AgeAdlt:SI130 -2.645e-01 6.887e-01 4.395e+02 -0.384 0.70108

AgepAdolesc:SI140 2.527e-02 6.578e-01 4.389e+02 0.038 0.96938

AgeAdolesc:SI140 2.780e-01 6.789e-01 4.390e+02 0.409 0.68240

AgeAdlt:SI140 -1.828e-02 6.887e-01 4.395e+02 -0.027 0.97883

AgepAdolesc:SI150 -1.906e-01 6.578e-01 4.389e+02 -0.290 0.77218

AgeAdolesc:SI150 3.676e-01 6.789e-01 4.390e+02 0.541 0.58851

AgeAdlt:SI150 1.858e-01 6.887e-01 4.395e+02 0.270 0.78747

SI100:HemisR 4.522e-02 4.786e-01 4.389e+02 0.094 0.92477

SI110:HemisR -1.507e-01 4.638e-01 4.389e+02 -0.325 0.74540

SI120:HemisR 1.805e-01 4.626e-01 4.389e+02 0.390 0.69657

SI130:HemisR 2.139e-02 4.626e-01 4.389e+02 0.046 0.96313

SI140:HemisR -9.823e-02 4.626e-01 4.389e+02 -0.212 0.83193

SI150:HemisR -2.135e-01 4.626e-01 4.389e+02 -0.461 0.64468

---

Signif. codes: 0 ‘***’ 0.001 ‘**’ 0.01 ‘*’ 0.05 ‘.’ 0.1 ‘ ’ 1

===========================================================================

Investigating feature: NT of ECR

===========================================================================

Linear mixed model fit by maximum likelihood . t-tests use Satterthwaite's method ['lmerModLmerTest']

Formula: curFormula

Data: TMSdev

AIC BIC logLik deviance df.resid

918.7 1085.4 -419.4 838.7 436

Scaled residuals:

Min 1Q Median 3Q Max

-3.2422 -0.6270 -0.0794 0.5581 4.5601

Random effects:

Groups Name Variance Std.Dev.

ID (Intercept) 0.1816 0.4262

Residual 0.2866 0.5353

Number of obs: 476, groups: ID, 38

Fixed effects:

Estimate Std. Error df t value Pr(>|t|)

(Intercept) 2.788605 0.251275 157.909349 11.098 < 2e-16 ***

AgepAdolesc -0.183068 0.316160 140.319936 -0.579 0.56349

AgeAdolesc 0.069495 0.323491 160.882439 0.215 0.83017

AgeAdlt 0.234205 0.328439 157.599585 0.713 0.47685

SI100 0.145605 0.250491 438.910049 0.581 0.56135

SI110 0.210199 0.244603 439.337574 0.859 0.39062

SI120 0.514719 0.244502 439.332491 2.105 0.03584 *

SI130 0.452894 0.244502 439.332491 1.852 0.06465 .

SI140 0.608553 0.244502 439.332491 2.489 0.01318 *

SI150 0.822134 0.244502 439.332491 3.362 0.00084 ***

HemisR -0.140664 0.192103 445.117999 -0.732 0.46441

AgepAdolesc:HemisR -0.010445 0.151783 445.721857 -0.069 0.94517

AgeAdolesc:HemisR -0.033743 0.150173 445.988001 -0.225 0.82232

AgeAdlt:HemisR -0.340836 0.154901 448.044388 -2.200 0.02829 *

AgepAdolesc:SI100 0.159342 0.300629 438.680824 0.530 0.59636

AgeAdolesc:SI100 -0.256891 0.308764 439.916190 -0.832 0.40586

AgeAdlt:SI100 -0.019047 0.313202 439.962230 -0.061 0.95153

AgepAdolesc:SI110 -0.016487 0.292696 439.709873 -0.056 0.95511

AgeAdolesc:SI110 -0.009575 0.303555 440.231281 -0.032 0.97485

AgeAdlt:SI110 0.063886 0.306396 440.584112 0.209 0.83493

AgepAdolesc:SI120 -0.242610 0.292695 439.709873 -0.829 0.40762

AgeAdolesc:SI120 -0.097388 0.302084 439.888792 -0.322 0.74731

AgeAdlt:SI120 -0.113900 0.306395 440.584158 -0.372 0.71026

AgepAdolesc:SI130 -0.073929 0.292695 439.709873 -0.253 0.80071

AgeAdolesc:SI130 0.266475 0.302084 439.888792 0.882 0.37819

AgeAdlt:SI130 0.161458 0.306395 440.584157 0.527 0.59849

AgepAdolesc:SI140 -0.053290 0.292695 439.709873 -0.182 0.85561

AgeAdolesc:SI140 0.244295 0.302084 439.888792 0.809 0.41912

AgeAdlt:SI140 0.193480 0.306395 440.584158 0.631 0.52806

AgepAdolesc:SI150 -0.068159 0.292695 439.709873 -0.233 0.81597

AgeAdolesc:SI150 0.337700 0.302084 439.888792 1.118 0.26422

AgeAdlt:SI150 0.062228 0.306395 440.584157 0.203 0.83915

SI100:HemisR 0.105002 0.212947 439.745524 0.493 0.62220

SI110:HemisR 0.190612 0.206370 439.775852 0.924 0.35618

SI120:HemisR 0.230818 0.205834 439.749777 1.121 0.26274

SI130:HemisR 0.313105 0.205834 439.749777 1.521 0.12894

SI140:HemisR 0.282510 0.205834 439.749777 1.373 0.17060

SI150:HemisR 0.293085 0.205834 439.749777 1.424 0.15519

---

Signif. codes: 0 ‘***’ 0.001 ‘**’ 0.01 ‘*’ 0.05 ‘.’ 0.1 ‘ ’ 1

===========================================================================

Investigating feature: Amp of FCR

===========================================================================

Linear mixed model fit by maximum likelihood . t-tests use Satterthwaite's method ['lmerModLmerTest']

Formula: curFormula

Data: TMSdev

AIC BIC logLik deviance df.resid

28.3 194.0 25.8 -51.7 425

Scaled residuals:

Min 1Q Median 3Q Max

-2.5873 -0.6290 -0.0161 0.6108 3.2520

Random effects:

Groups Name Variance Std.Dev.

ID (Intercept) 0.02881 0.1697

Residual 0.04382 0.2093

Number of obs: 465, groups: ID, 38

Fixed effects:

Estimate Std. Error df t value Pr(>|t|)

(Intercept) 1.95165 0.09552 132.02214 20.431 < 2e-16 ***

AgepAdolesc 0.11887 0.12401 129.47773 0.959 0.339596

AgeAdolesc 0.11577 0.12656 147.97021 0.915 0.361813

AgeAdlt 0.01193 0.12630 136.90585 0.094 0.924870

SI100 0.08141 0.09544 426.89296 0.853 0.394187

SI110 0.14093 0.09269 426.52538 1.520 0.129152

SI120 0.28479 0.09260 426.46509 3.076 0.002237 **

SI130 0.33863 0.09255 426.44553 3.659 0.000285 ***

SI140 0.40289 0.09255 426.44553 4.353 1.68e-05 ***

SI150 0.44180 0.09266 426.52765 4.768 2.56e-06 ***

HemisR 0.14695 0.07384 431.65531 1.990 0.047197 *

AgepAdolesc:HemisR -0.06881 0.05937 432.82574 -1.159 0.247148

AgeAdolesc:HemisR -0.26253 0.05936 434.12304 -4.423 1.23e-05 ***

AgeAdlt:HemisR -0.15814 0.06070 435.57717 -2.605 0.009490 **

AgepAdolesc:SI100 0.06749 0.11775 426.75378 0.573 0.566833

AgeAdolesc:SI100 0.08948 0.12074 428.14594 0.741 0.459031

AgeAdlt:SI100 0.15928 0.11955 428.04367 1.332 0.183453

AgepAdolesc:SI110 0.15894 0.11473 427.35175 1.385 0.166657

AgeAdolesc:SI110 0.18038 0.11787 428.12364 1.530 0.126665

AgeAdlt:SI110 0.26018 0.11735 428.58810 2.217 0.027144 *

AgepAdolesc:SI120 0.13406 0.11433 427.67259 1.173 0.241635

AgeAdolesc:SI120 0.27834 0.11725 427.54393 2.374 0.018042 *

AgeAdlt:SI120 0.35821 0.11735 428.58687 3.052 0.002411 **

AgepAdolesc:SI130 0.23261 0.11433 427.67258 2.035 0.042513 *

AgeAdolesc:SI130 0.42759 0.11725 427.54355 3.647 0.000298 ***

AgeAdlt:SI130 0.43651 0.11655 428.12439 3.745 0.000205 ***

AgepAdolesc:SI140 0.23517 0.11433 427.67258 2.057 0.040300 *

AgeAdolesc:SI140 0.44094 0.11725 427.54355 3.761 0.000193 ***

AgeAdlt:SI140 0.48011 0.11655 428.12439 4.119 4.56e-05 ***

AgepAdolesc:SI150 0.25585 0.11433 427.67266 2.238 0.025747 *

AgeAdolesc:SI150 0.45626 0.11790 427.73875 3.870 0.000126 ***

AgeAdlt:SI150 0.51844 0.11735 428.58802 4.418 1.26e-05 ***

SI100:HemisR -0.05316 0.08353 427.47617 -0.636 0.524830

SI110:HemisR 0.09482 0.08098 427.48654 1.171 0.242316

SI120:HemisR 0.08410 0.08049 427.15333 1.045 0.296677

SI130:HemisR 0.08069 0.08021 427.04871 1.006 0.315031

SI140:HemisR 0.04125 0.08021 427.04871 0.514 0.607308

SI150:HemisR 0.03562 0.08082 427.50493 0.441 0.659598

---

Signif. codes: 0 ‘***’ 0.001 ‘**’ 0.01 ‘*’ 0.05 ‘.’ 0.1 ‘ ’ 1

===========================================================================

Investigating feature: iDur of FCR

===========================================================================

Linear mixed model fit by maximum likelihood . t-tests use Satterthwaite's method ['lmerModLmerTest']

Formula: curFormula

Data: TMSdev

AIC BIC logLik deviance df.resid

2929.5 3095.2 -1424.7 2849.5 425

Scaled residuals:

Min 1Q Median 3Q Max

-2.9746 -0.5736 -0.0402 0.5153 2.6527

Random effects:

Groups Name Variance Std.Dev.

ID (Intercept) 12.34 3.513

Residual 22.76 4.771

Number of obs: 465, groups: ID, 38

Fixed effects:

Estimate Std. Error df t value Pr(>|t|)

(Intercept) 18.4615 2.0894 154.0364 8.836 2.11e-15 ***

AgepAdolesc 2.0500 2.7111 150.6890 0.756 0.450751

AgeAdolesc 5.7164 2.7759 172.8624 2.059 0.040966 *

AgeAdlt 1.8234 2.7649 159.2656 0.659 0.510541

SI100 2.9606 2.1751 427.6540 1.361 0.174194

SI110 3.4217 2.1125 427.2233 1.620 0.106031

SI120 5.7844 2.1104 427.1553 2.741 0.006383 **

SI130 5.7081 2.1092 427.1327 2.706 0.007076 **

SI140 8.5271 2.1092 427.1327 4.043 6.26e-05 ***

SI150 9.6112 2.1118 427.2267 4.551 6.96e-06 ***

HemisR 4.8669 1.6819 433.0362 2.894 0.003999 **

AgepAdolesc:HemisR -2.1982 1.3522 434.3376 -1.626 0.104757

AgeAdolesc:HemisR -6.3496 1.3517 435.7815 -4.697 3.54e-06 ***

AgeAdlt:HemisR -3.6736 1.3819 437.4008 -2.658 0.008140 **

AgepAdolesc:SI100 0.5049 2.6835 427.4927 0.188 0.850861

AgeAdolesc:SI100 -2.0349 2.7513 429.0825 -0.740 0.459937

AgeAdlt:SI100 1.3271 2.7242 428.9751 0.487 0.626407

AgepAdolesc:SI110 2.5439 2.6144 428.1869 0.973 0.331087

AgeAdolesc:SI110 -0.7622 2.6857 429.0532 -0.284 0.776692

AgeAdlt:SI110 3.3758 2.6740 429.6053 1.262 0.207459

AgepAdolesc:SI120 2.0625 2.6053 428.5580 0.792 0.429017

AgeAdolesc:SI120 1.0387 2.6719 428.4038 0.389 0.697648

AgeAdlt:SI120 5.8057 2.6739 429.6039 2.171 0.030461 *

AgepAdolesc:SI130 5.1281 2.6053 428.5580 1.968 0.049677 *

AgeAdolesc:SI130 3.5910 2.6718 428.4034 1.344 0.179651

AgeAdlt:SI130 9.4594 2.6558 429.0725 3.562 0.000410 ***

AgepAdolesc:SI140 4.8063 2.6053 428.5580 1.845 0.065756 .

AgeAdolesc:SI140 4.5539 2.6718 428.4034 1.704 0.089030 .

AgeAdlt:SI140 9.5533 2.6558 429.0725 3.597 0.000359 ***

AgepAdolesc:SI150 4.4805 2.6053 428.5581 1.720 0.086202 .

AgeAdolesc:SI150 4.4423 2.6867 428.6406 1.653 0.098976 .

AgeAdlt:SI150 7.8331 2.6739 429.6052 2.929 0.003577 **

SI100:HemisR -1.8578 1.9036 428.3146 -0.976 0.329641

SI110:HemisR 1.2952 1.8455 428.3270 0.702 0.483155

SI120:HemisR 1.0376 1.8342 427.9517 0.566 0.571901

SI130:HemisR 1.5980 1.8280 427.8308 0.874 0.382491

SI140:HemisR -0.5565 1.8280 427.8308 -0.304 0.760962

SI150:HemisR 0.7830 1.8417 428.3529 0.425 0.670926

---

Signif. codes: 0 ‘***’ 0.001 ‘**’ 0.01 ‘*’ 0.05 ‘.’ 0.1 ‘ ’ 1

===========================================================================

Investigating feature: Lat of FCR

===========================================================================

Linear mixed model fit by maximum likelihood . t-tests use Satterthwaite's method ['lmerModLmerTest']

Formula: curFormula

Data: TMSdev

AIC BIC logLik deviance df.resid

1262.3 1428.0 -591.1 1182.3 425

Scaled residuals:

Min 1Q Median 3Q Max

-3.2435 -0.6128 -0.0538 0.5604 3.2395

Random effects:

Groups Name Variance Std.Dev.

ID (Intercept) 0.4640 0.6811

Residual 0.6163 0.7851

Number of obs: 465, groups: ID, 38

Fixed effects:

Estimate Std. Error df t value Pr(>|t|)

(Intercept) 18.65905 0.36981 123.76936 50.456 < 2e-16 ***

AgepAdolesc -1.41169 0.48026 121.62073 -2.939 0.003936 **

AgeAdolesc -0.08884 0.48895 138.12614 -0.182 0.856082

AgeAdlt 0.65238 0.48863 128.41107 1.335 0.184196

SI100 -0.45390 0.35799 428.37949 -1.268 0.205517

SI110 -0.76417 0.34766 428.06702 -2.198 0.028482 *

SI120 -1.05738 0.34730 428.01452 -3.045 0.002474 **

SI130 -1.20920 0.34711 427.99775 -3.484 0.000546 ***

SI140 -1.75384 0.34711 427.99775 -5.053 6.46e-07 ***

SI150 -1.81641 0.34754 428.06858 -5.226 2.71e-07 ***

HemisR -0.78999 0.27703 432.51834 -2.852 0.004558 **

AgepAdolesc:HemisR 0.61814 0.22278 433.54499 2.775 0.005765 **

AgeAdolesc:HemisR 0.76252 0.22275 434.68236 3.423 0.000678 ***

AgeAdlt:HemisR 0.09170 0.22779 435.95632 0.403 0.687474

AgepAdolesc:SI100 0.17437 0.44164 428.26030 0.395 0.693167

AgeAdolesc:SI100 0.74326 0.45291 429.46309 1.641 0.101517

AgeAdlt:SI100 0.06395 0.44844 429.37015 0.143 0.886674

AgepAdolesc:SI110 0.23499 0.43032 428.77153 0.546 0.585283

AgeAdolesc:SI110 0.48589 0.44212 429.44563 1.099 0.272378

AgeAdlt:SI110 0.22939 0.44021 429.83628 0.521 0.602572

AgepAdolesc:SI120 0.44578 0.42885 429.04642 1.039 0.299170

AgeAdolesc:SI120 0.85776 0.43979 428.93882 1.950 0.051780 .

AgeAdlt:SI120 0.23151 0.44020 429.83521 0.526 0.599211

AgepAdolesc:SI130 0.36629 0.42885 429.04642 0.854 0.393509

AgeAdolesc:SI130 0.49831 0.43978 428.93849 1.133 0.257815

AgeAdlt:SI130 0.16489 0.43719 429.43739 0.377 0.706245

AgepAdolesc:SI140 0.46591 0.42885 429.04642 1.086 0.277906

AgeAdolesc:SI140 0.67935 0.43978 428.93849 1.545 0.123148

AgeAdlt:SI140 0.44161 0.43719 429.43739 1.010 0.313015

AgepAdolesc:SI150 0.46869 0.42885 429.04648 1.093 0.275049

AgeAdolesc:SI150 0.68197 0.44225 429.10033 1.542 0.123799

AgeAdlt:SI150 0.35653 0.44021 429.83620 0.810 0.418445

SI100:HemisR 0.05365 0.31332 428.88586 0.171 0.864120

SI110:HemisR 0.09338 0.30376 428.89462 0.307 0.758668

SI120:HemisR -0.24465 0.30190 428.60425 -0.810 0.418166

SI130:HemisR -0.36405 0.30086 428.51451 -1.210 0.226926

SI140:HemisR -0.08900 0.30086 428.51451 -0.296 0.767509

SI150:HemisR -0.14843 0.30314 428.90815 -0.490 0.624646

---

Signif. codes: 0 ‘***’ 0.001 ‘**’ 0.01 ‘*’ 0.05 ‘.’ 0.1 ‘ ’ 1

===========================================================================

Investigating feature: T1T of FCR

===========================================================================

Linear mixed model fit by maximum likelihood . t-tests use Satterthwaite's method ['lmerModLmerTest']

Formula: curFormula

Data: TMSdev

AIC BIC logLik deviance df.resid

1534.3 1700.0 -727.2 1454.3 425

Scaled residuals:

Min 1Q Median 3Q Max

-3.1649 -0.6342 -0.1194 0.6074 3.2527

Random effects:

Groups Name Variance Std.Dev.

ID (Intercept) 1.046 1.023

Residual 1.086 1.042

Number of obs: 465, groups: ID, 38

Fixed effects:

Estimate Std. Error df t value Pr(>|t|)

(Intercept) 23.13726 0.52317 102.89269 44.225 < 2e-16 ***

AgepAdolesc -1.11399 0.67983 101.38606 -1.639 0.10439

AgeAdolesc -0.03409 0.68913 114.02719 -0.049 0.96063

AgeAdlt -0.05404 0.69035 106.76747 -0.078 0.93776

SI100 -0.19381 0.47530 427.58693 -0.408 0.68364

SI110 -0.35528 0.46157 427.33438 -0.770 0.44190

SI120 -0.88432 0.46110 427.29031 -1.918 0.05579 .

SI130 -0.52545 0.46083 427.27657 -1.140 0.25483

SI140 -0.91495 0.46083 427.27657 -1.985 0.04774 *

SI150 -1.24514 0.46142 427.33514 -2.699 0.00724 **

HemisR -0.02510 0.36800 431.05428 -0.068 0.94565

AgepAdolesc:HemisR 0.40261 0.29598 431.92758 1.360 0.17445

AgeAdolesc:HemisR 0.08228 0.29598 432.89538 0.278 0.78115

AgeAdlt:HemisR -0.67924 0.30273 433.97862 -2.244 0.02535 *

AgepAdolesc:SI100 0.41818 0.58636 427.48936 0.713 0.47612

AgeAdolesc:SI100 0.48787 0.60141 428.48737 0.811 0.41770

AgeAdlt:SI100 0.23802 0.59546 428.40402 0.400 0.68956

AgepAdolesc:SI110 -0.25689 0.57136 427.90633 -0.450 0.65322

AgeAdolesc:SI110 0.07169 0.58708 428.47525 0.122 0.90286

AgeAdlt:SI110 0.64720 0.58458 428.78520 1.107 0.26886

AgepAdolesc:SI120 0.28429 0.56943 428.13133 0.499 0.61786

AgeAdolesc:SI120 0.36269 0.58395 428.04685 0.621 0.53486

AgeAdlt:SI120 0.82221 0.58456 428.78430 1.407 0.16029

AgepAdolesc:SI130 0.12769 0.56943 428.13132 0.224 0.82267

AgeAdolesc:SI130 -0.42008 0.58394 428.04658 -0.719 0.47230

AgeAdlt:SI130 0.62048 0.58053 428.45657 1.069 0.28575

AgepAdolesc:SI140 0.19757 0.56943 428.13132 0.347 0.72879

AgeAdolesc:SI140 -0.34557 0.58394 428.04658 -0.592 0.55431

AgeAdlt:SI140 0.95248 0.58053 428.45657 1.641 0.10159

AgepAdolesc:SI150 0.50805 0.56943 428.13137 0.892 0.37278

AgeAdolesc:SI150 -0.04293 0.58723 428.17193 -0.073 0.94176

AgeAdlt:SI150 1.13741 0.58457 428.78513 1.946 0.05234 .

SI100:HemisR -0.27022 0.41603 428.01020 -0.650 0.51634

SI110:HemisR 0.09775 0.40333 428.01721 0.242 0.80862

SI120:HemisR -0.07099 0.40084 427.77306 -0.177 0.85952

SI130:HemisR -0.46354 0.39945 427.69949 -1.160 0.24651

SI140:HemisR -0.26130 0.39945 427.69949 -0.654 0.51337

SI150:HemisR -0.25088 0.40250 428.02536 -0.623 0.53343

---

Signif. codes: 0 ‘***’ 0.001 ‘**’ 0.01 ‘*’ 0.05 ‘.’ 0.1 ‘ ’ 1

===========================================================================

Investigating feature: NT of FCR

===========================================================================

Linear mixed model fit by maximum likelihood . t-tests use Satterthwaite's method ['lmerModLmerTest']

Formula: curFormula

Data: TMSdev

AIC BIC logLik deviance df.resid

890.9 1056.6 -405.4 810.9 425

Scaled residuals:

Min 1Q Median 3Q Max

-2.5804 -0.7335 -0.0782 0.5779 3.6258

Random effects:

Groups Name Variance Std.Dev.

ID (Intercept) 0.07092 0.2663

Residual 0.29989 0.5476

Number of obs: 465, groups: ID, 38

Fixed effects:

Estimate Std. Error df t value Pr(>|t|)

(Intercept) 3.273387 0.210636 272.850190 15.540 < 2e-16 ***

AgepAdolesc -0.472566 0.272618 263.941175 -1.733 0.08419 .

AgeAdolesc -0.492523 0.282638 298.391308 -1.743 0.08243 .

AgeAdlt -0.216401 0.279290 274.059308 -0.775 0.43911

SI100 0.023253 0.249505 429.307072 0.093 0.92579

SI110 -0.014915 0.242382 428.460229 -0.062 0.95096

SI120 0.187679 0.242144 428.352539 0.775 0.43873

SI130 0.231348 0.242010 428.311578 0.956 0.33964

SI140 0.463774 0.242010 428.311578 1.916 0.05599 .

SI150 0.741459 0.242299 428.474805 3.060 0.00235 **

HemisR -0.248040 0.192371 437.892509 -1.289 0.19795

AgepAdolesc:HemisR 0.062165 0.154559 439.756275 0.402 0.68772

AgeAdolesc:HemisR -0.056339 0.154379 441.878153 -0.365 0.71533

AgeAdlt:HemisR 0.055452 0.157682 444.259648 0.352 0.72525

AgepAdolesc:SI100 -0.136556 0.307844 429.002910 -0.444 0.65756

AgeAdolesc:SI100 0.162031 0.315360 431.721246 0.514 0.60766

AgeAdlt:SI100 0.116694 0.312262 431.616284 0.374 0.70881

AgepAdolesc:SI110 0.098626 0.299804 430.295696 0.329 0.74234

AgeAdolesc:SI110 0.533105 0.307850 431.632071 1.732 0.08404 .

AgeAdlt:SI110 0.156129 0.306399 432.766530 0.510 0.61062

AgepAdolesc:SI120 -0.168289 0.298704 430.971811 -0.563 0.57346

AgeAdolesc:SI120 0.422292 0.306358 430.647867 1.378 0.16879

AgeAdlt:SI120 0.152028 0.306392 432.764329 0.496 0.62001

AgepAdolesc:SI130 -0.244650 0.298703 430.971768 -0.819 0.41322

AgeAdolesc:SI130 0.539851 0.306356 430.647080 1.762 0.07875 .

AgeAdlt:SI130 0.283080 0.304407 431.835380 0.930 0.35292

AgepAdolesc:SI140 -0.320615 0.298703 430.971768 -1.073 0.28371

AgeAdolesc:SI140 0.603670 0.306356 430.647080 1.970 0.04942 *

AgeAdlt:SI140 0.237982 0.304407 431.835380 0.782 0.43477

AgepAdolesc:SI150 -0.386641 0.298704 430.972017 -1.294 0.19622

AgeAdolesc:SI150 0.384677 0.308019 431.191711 1.249 0.21239

AgeAdlt:SI150 0.049279 0.306396 432.766547 0.161 0.87230

SI100:HemisR 0.028825 0.218280 430.375372 0.132 0.89500

SI110:HemisR 0.095741 0.211616 430.402592 0.452 0.65119

SI120:HemisR 0.078453 0.210369 429.814457 0.373 0.70938

SI130:HemisR 0.068836 0.209662 429.596644 0.328 0.74283

SI140:HemisR 0.066913 0.209662 429.596644 0.319 0.74977

SI150:HemisR -0.001404 0.211176 430.497106 -0.007 0.99470

---

Signif. codes: 0 ‘***’ 0.001 ‘**’ 0.01 ‘*’ 0.05 ‘.’ 0.1 ‘ ’ 1
